# Ontogenetic stage and type of donor cells shape extracellular vesicles’ therapeutic potential for osteoarthritis

**DOI:** 10.1186/s13287-025-04585-y

**Published:** 2025-09-01

**Authors:** K. Tarasova, M.B. Arteaga, A. Kidtiwong, H. Nivarthi, J. Gamauf, G. Corso, S. Gültekin, A. Bileck, M. Rothbauer, S. Toegel, M. Hackl, S. Kau-Strebinger, C. Gerner, R. Grillari, I. Gerner, F. Jenner

**Affiliations:** 1 Department for Small Animals and Horses, Centre for Equine Health and Research, Equine Surgery Unit, Veterinary Tissue Engineering and Regenerative Medicine Laboratory, University of Veterinary Medicine Vienna, Vienna, Austria; 2 Evercyte GmbH, Vienna, Austria; 3 Department of Analytical Chemistry, University Vienna, Vienna, Austria; 4 Department of Orthopedics and Trauma Surgery, Karl Chiari Lab for Orthopaedic Biology, Medical University Vienna, Vienna, Austria; 5 TAmiRNA GmbH, Vienna, Austria; 6 Department of Biological Sciences and Pathobiology, Centre of Pathobiology, Morphology Unit, University of Veterinary Medicine Vienna, Vienna, Austria; 7 Austrian Cluster for Tissue Regeneration, Vienna, Austria

**Keywords:** Extracellular vesicle, Chondrocyte, Synoviocytes, Fetal, Perinatal, Osteoarthritis, Donor age

## Abstract

**Background:**

Osteoarthritis (OA) remains an intractable condition due to the limited regenerative capacity of adult cartilage. Extracellular vesicles (EVs) have emerged as promising therapeutics, yet the optimal donor cell source is still undetermined, as both donor cell type and age significantly influence EV therapeutic efficacy. This study evaluates the therapeutic potential of EVs derived from ovine fetal articular chondrocytes (fCCs) and ovine fetal umbilical cord blood mesenchymal stromal cells (fMSCs) compared to EVs from two immortalized human perinatal cell lines, Wharton’s jelly (WJ-MSCs) and amnion MSCs (P-MSCs), on inflamed ovine adult chondrocytes and synoviocytes in vitro.

**Methods:**

EVs were isolated from conditioned media using tangential flow filtration and characterized by size, concentration, and EV markers. Inflamed adult articular chondrocytes and synoviocytes were treated with 1E + 09 particles/mL of each EV source. EV’s cellular uptake was assessed via live-cell imaging, flow cytometry, and confocal microscopy. Therapeutic effects were evaluated through proliferation, wound healing assays, and multi-omics (RNASeq, proteomics) analyses at 24 and 48 h post-treatment.

**Results:**

All EVs were successfully internalized by inflamed ovine and human chondrocytes. Donor cell type significantly influenced incorporation with fCC-EVs achieving the highest uptake across conditions. All treatments reduced pro-inflammatory genes and upregulated growth and cell cycle-related genes. Fetal-derived EVs induced more robust transcriptional changes and enriched signaling pathways than perinatal-derived EVs. Notably, fCC-EVs exhibited the most pronounced effects on inflamed chondrocytes, while fMSC-EVs were most effective on synoviocytes. Donor cell age emerged as a more influential factor in therapeutic efficacy than cell type.

**Conclusions:**

The ontogenetic stage of donor cells plays a crucial role in EV’s therapeutic efficacy, with fetal-derived EVs demonstrating superior outcomes compared to perinatal-derived EVs. The distinct effects of fCC-EVs and fMSC-EVs suggest that a combinatorial approach using both EV types could optimize therapeutic outcomes.

**Supplementary Information:**

The online version contains supplementary material available at 10.1186/s13287-025-04585-y.

## Background

Osteoarthritis (OA) is the most common chronic musculoskeletal disease and a leading cause of disability [1, 2]. Characterized by a progressive loss of articular cartilage integrity, synovial inflammation, and subchondral bone remodeling, OA manifests clinically through pain, stiffness, and functional limitations, significantly compromising patients’ quality of life [1, 3–6]. To date, therapeutic interventions capable of halting the degradation of articular cartilage in OA and restoring its original mechanical and functional integrity remain elusive. Consequently, OA’s high global prevalence of 22.9% in persons over 40 years of age (corresponding to 654.1 million individuals) poses a significant health and welfare problem, underscoring the urgent need for effective interventions [1, 3–6]. 

Due to the complex molecular derangements in OA, which entail a vicious cycle of extracellular matrix (ECM) degradation and articular inflammation, effective OA therapy necessitates a comprehensive approach targeting not only inflammation but also chondrocyte phenotype stability, cellular senescence, and ECM production to promote joint homeostasis and alleviate pain.

Intra-articular injection of mesenchymal stromal cells (MSCs) has emerged as a promising therapeutic approach for both isolated cartilage defects and OA, demonstrating potential to improve clinical outcomes, reduce inflammation, and alleviate pain [7–10]. However, MSCs efficacy diminishes with advancing age and disease progression [11–18], precisely when the need for regenerative therapies increases. Furthermore, MSCs exhibit phenotypic instability during in vitro expansion, characterized by replicative senescence, reduced immunomodulatory properties, and a shift from a pro-regenerative to a pro-inflammatory profile [19–24]. Since MSCs exert their therapeutic effects largely through paracrine mechanisms, the extracellular vesicles (EVs) they secrete offer an attractive alternative. EVs, which mirror the bioactivity and therapeutic potential of their parent cells, have demonstrated equivalent therapeutic potential in treating various conditions, including OA [25–30]. Several in vitro and in vivo studies have demonstrated the chondroprotective effects of EVs derived from MSCs under inflammatory osteoarthritic conditions [31–34]. In vitro, IL-1β (1 ng/mL, 24 h) stimulation of osteoarthritic chondrocytes followed by treatment with hBMSC-EVs (10 µg/mL, 24 h) enhanced proliferation, reduced apoptosis, and restored migration [31]. This was accompanied by increased expression of anabolic genes (PRG4, ACAN, COL2A1, SOX9, BCL2, COMP) and downregulation of catabolic and inflammatory genes (IL-1B, MMP13, ALPL, COL10A1) as well as reduced activation of ERK1/2, PI3K/Akt, p38, TAK1, and NF-κB signaling pathways [31]. Similarly, murine chondrocytes treated with IL-1β (1 ng/mL) and exposed to BM-MSC-derived exosomes or microvesicles (12.5 ng–1.25 µg) showed restored COL2A1 and ACAN expression and suppressed NOS2, MMP13, and ADAMTS5, indicating anti-inflammatory and anabolic effects [32]. In temporomandibular joint OA (TMJOA) models, both in vitro (2–4 × 10⁸ BMSC-sEVs/well) and in vivo (200 µL of 4–8 × 10⁸ sEVs/joint) BMSC-sEVs attenuated inflammation-induced cartilage damage and promoted regeneration in a dose-dependent manner [33]. Likewise, intra-articular administration of hucMSC-EVs (10 µL, 1 × 10¹¹ particles/mL, twice weekly) in destabilization of the medial meniscus (DMM)-induced OA mice improved cartilage structure, reduced osteophyte formation, and modulated gene expression by suppressing MMP13 and ADAMTS5, while enhancing COL2A1 and ACAN expression [34]. 

As key mediators of intercellular communication, EVs transfer their bioactive cargo, including proteins, lipids, and nucleic acids, from donor to recipient cells, inducing transcriptional reprogramming and epigenetic modifications that regulate cellular function, signaling and phenotype [35–42]. Notably, EVs injected into the knee joint can penetrate into the deep zone of articular cartilage [43–45], where they are internalized by chondrocytes, promoting cartilage regeneration, reducing the inflammatory response in OA chondrocytes and stimulating ECM production [27, 32, 43–45]. 

As EVs exert comparable therapeutic effects as their parent cells, identifying the optimal cell source for EV production is crucial. The cargo composition and surface molecular profile of EVs is determined by the characteristics of the donor cells, including their tissue provenance, developmental stage, lineage, differentiation status, and microenvironment [35–37, 46, 47]. In addition, the age of the parental cell plays a significant role, as EVs from senescent cells exhibit pro-catabolic and pro-inflammatory effects, whereas those from young donors in early passages demonstrate chondroprotective and rejuvenating properties [46, 48–58]. This highlights the limitations of autologous transplantation and underscores the importance of careful donor and cell selection to optimize EV therapy. The ability of EVs to mediate horizontal transfer of functional molecules across species barriers, as evidenced by the production of mouse proteins in human mast cells following the transfer of mouse mRNA via EVs [28], supports the viability of xenogeneic administration of EVs from optimized cell sources, thereby expanding the range of eligible EV donor cells.

Given that perinatal MSCs, such as Wharton’s Jelly, umbilical cord blood or amnion derived MSCs, have demonstrated superior anti-inflammatory and immunosuppressive properties compared to MSCs from adult tissues [56, 59–65], juvenile cells represent a promising EV source for regenerative applications.

Similarly, the superior chondrogenic potential of articular progenitor cells compared to those from non-articular tissues suggests their suitability as EV donors for cartilage repair and regeneration therapies [30, 66–70]. 

Therefore, this study compares the therapeutic potential of EVs derived from ovine fetal articular chondrocytes, ovine fetal umbilical cord blood derived MSCs, human perinatal Wharton’s jelly- and placental amnion-derived MSCs in an in vitro ovine model of OA.

## Methods

### EV donor cell culture for conditioned medium production

EVs from four different donor cell populations were compared: two primary ovine fetal (day 80–89 gestation) cell types, articular chondrocytes (fCCs) and umbilical cord blood-derived MSCs (fMSCs), and two telomerized human perinatal cell lines, Wharton’s Jelly-derived MSCs (WJ-MSC/TERT273, Evercyte GmbH, Vienna, Austria, Cat# CHT-021-0273) and placental amnion-derived MSCs (P-MSC/TERT308, Evercyte GmbH, Cat# CHT-051-0308). Primary ovine cells (*n* = 4 biological replicates) had been isolated and biobanked from sheep euthanized for reasons unrelated to this study as previously described [68,69] and were used in passage 3. The work has been reported in line with the ARRIVE guidelines 2.0.

Ovine and human cells were expanded in monolayer culture before transfer to a hollow fiber bioreactor (HFB, Fibercell Systems Inc, USA, Cat# C2011 and C2025D) for conditioned medium (CM) production to facilitate continuous long-term culture without passaging. In the HFB, the capillaries containing the full medium with supplements are separated from the extracapillary space (ECS) containing the cells by a membrane (20 kDa cut-off), which allows only nutrients for the cells but no EVs to pass through, thereby ensuring that the obtained EVs derive solely from the cells seeded into the ECS and not the media supplements.

The bioreactor was set up according to the manufacturer’s instructions. First, the PBS -/- (Mg2+/Ca2+, Gibco, Thermo Fisher Scientific, USA) was filtered with a 0.22 μm Polyethersulfone (PES, Thermofisher Scientific, USA, 566 − 0020), then the cartridge was equilibrated with 400mL of filtered PBS -/- (fPBS) for 24 h at a pumping rate of 20 (80 mL/min). Afterwards the cartridge was equilibrated with 150mL of xeno-free basal media (fetal cells: StemMACS^®^, Miltenyi Biotech; perinatal cells: WJ-MSC/TERT273: MesenCult^TM^-ACF Plus Medium, Stemcell Technologies Canada Inc., Canada, P-MSC/TERT308: MSC NutriStem^®^XF Medium, Satorius AG, Germany) at a pumping rate of 20–25 (80–100 mL/min) for 24 h and finally with xeno-free basal medium with supplements (fetal cells StemMACS XF and 1% Pen/Strep ; perinatal cells WJ-MSC/TERT273: MesenCult^TM^-ACF Plus 500X Supplement, P-MSC/TERT308: MSC NutriStem^®^SX Supplement Mix) at a pumping rate of 20–25 (80–100 mL/min) for 24 h. The ECS was flushed and filled with basal medium without supplements. Fetal cells were seeded without prior coating (fCCs: 1.65E + 06cells/small bioreactor − 9.70E + 07 cells/medium bioreactor; fMSCs: 1.26E + 06/small bioreactor − 9.50E + 07 cells/medium bioreactor), whereas for perinatal cells the ECS was coated before seeding (WJ-MSC/TERT273: 4,80E + 7 − 1,09E + 8 cells/medium reactor, P-MSC/TERT308: 1.02E + 7 cells/small reactor). For WJ-MSC/TERT273 fibers were coated as recommended by the manufacturer with Animal Component-Free Cell Attachment Substrate, Stemcell Technologies Canada Inc. two hours at room temperature, while for P-MSC/TERT308 fibers were coated for one hour at RT with Vitronectin (VTN-N) Recombinant Human Protein, Truncated (Thermo Fisher Scientific) diluted 1:100 in PBS. Coated fibers were rinsed with basal medium and cells seeded into ECS compartments.

Starting one week after seeding, the CM from fetal ovine derived EVs was collected daily, centrifuged 10 min x 2000 g at 4 °C and stored at -80 °C until further use.

Prior to seeding in the HFB, MSC phenotype of WJ-MSC/TERT273 and P-MSC/TERT308 was characterized by flow cytometry using MSCs markers - CD73, CD105 and CD90- and CD34 as hematopoietic progenitor cell marker (See Suppl. Materials). Furthermore, to confirm phenotype maintenance of the primary ovine cells, which were expected to be potentially less stable than the immortalized perinatal cell lines, throughout 6 weeks culture, the cells were aseptically harvested and characterized according to the criteria of the International Society of Cell and Gene Therapy [71], using tri-lineage differentiation and flow cytometry for fMSCs, and alcian blue staining for fCCs (see Suppl. Materials).

### EV isolation and characterization

EVs were enriched from CM by tangential flow filtration (300 kDa cut off, Repligen, USA, C02-E300-05-S and D06-E300-05-S) following the manufacturer’s instructions. Fetal and perinatal-derived EVs were then characterized for particle size distribution and number by nanoparticle tracking analysis (NTA), and morphology by transmission electron microscopy. Presence of marker proteins (tetraspanins: CD9, CD63, CD81) was assessed by beads-based flow cytometry for human perinatal-derived EVs, whereas tetraspanins and non-EV (Calnexin) markers were analysed by Western Blotting for ovine fetal cell-derived EVs. Moreover, ovine fetal EVs were also analysed by fluorescence-triggered flow cytometry (FT-FC). EVs derived from primary cells (fCC and fMSCs) were pooled from 4 donors to achieve sufficient quantities for all experiments. The same pool of EVs was used for all experiments involving primary cell-derived EVs. All EV preparations were aliquoted and stored in a 20mM HEPES solution at − 80 °C until further use.

The production, isolation, and characterization of EVs were carried out in alignment with the MISEV2023 guidelines to the extent feasible [72]. However, due to the lack of cross-reacting antibodies available for Fluorescence-Triggered Flow Cytometry (FT-FC) of ovine samples a complete alignment of characterization methods was not possible.

### Nanoparticle Tracking Analysis (NTA)

Fetal derived EVs isolates were diluted in filtered PBS and analysed using a ZetaView^®^ TWIN x20 System (Particle Metrix GmbH, Germany) and the perinatal derived EVs were analysed using ZetaView BASIC PMX-120 (Particle Metrix GmbH, Germany). The ZetaView device was calibrated using the provided standard beads. Measurements were performed in scatter mode with a 488 nm laser. Technical triplicates were conducted for each biological replicate to ensure reproducibility. The instrument settings were as follows: Minimum brightness threshold of 30, maximum brightness of 255, minimum area of 10 pixels, maximum area of 5000 pixels, the sensitivity was set at 80, the shutter was set at 100, temperature was set at 23–25 °C C. Data acquisition and .txt export was performed using the ZetaView software version 8.06.01 SP1 (Particle Metrix GmbH, Germany). Exported data were analysed in Excel (Microsoft Inc., USA) and displayed in GraphPad Prism (version 10.2.3).

### CMG labelling - based Fluorescence-Triggered Flow Cytometry (FT-FC) for characterization of fetal derived EVs

To verify the presence of lipid membrane-based nanoparticles (EVs at high likelihood) and to approximate their size distribution according to predefined gates, a CytoFlexS flow cytometer and CytExpert software version 1.2 (Beckman Coulter, USA) were used according to a previously published fluorescence triggering protocol [73]. Briefly, this protocol utilizes a FITC-based fluorescence trigger instead of the conventional side scatter (SSC) trigger, enhancing the exclusion of nonspecific background and allowing for the detection of EVs in the sub-200 nm range while minimizing sample autofluorescence. Size gating was performed using signals from FITC-labeled silica beads (100 nm, 200 nm, and 500 nm; Kisker Biotech, Germany), defining gates as follows: i) EVs ≤ 200 nm, ii) > 200–500 nm, and iii) > 500 nm. For fluorescence triggering, fetal EVs were stained with the lipid membrane dye Cell Mask Green (CMG) (Thermo Fisher Scientific, USA). Specifically, 10 µL EV sample, adjusted to give a final concentration of 1E + 07 particles/mL, was mixed with 50 µL of CMG (1:2000 dilution in fresh 0.2 μm fPBS-/- and incubated for 30 min at 37 °C in the dark. Samples were then diluted to a total volume of 200 µL with 1x fPBS and either measured immediately or kept on ice until measurement. The flow cytometer was set to a sample flow rate of 30 µL/minute, with measurements continuing until over 10,000 events were recorded in the respective gates. Controls included 1x fPBS-/- with and w/o CMG, and EV samples w/o CMG. For lysis control, 100 µL of RIPA-Lysis buffer was added to CMG-stained samples and incubated for 30 min on ice before measurement. Data analysis was performed using FlowJo software version 10 (FlowJo LLC, USA).

### CD81 + beads-based Fluorescence-Triggered Flow Cytometry (FT-FC)

#### For characterization of perinatal derived EVs

The analysis of classical tetraspanin markers on WJ-MSC/TERT273 and P-MSC/TERT308-derived EVs was carried out using exosome-human CD81 beads (Invitrogen) to capture EVs, followed by antibody staining and flow cytometric analysis. Briefly, EV samples were diluted to a concentration of 1 × 10^9^ particles in 100 µL of filtered PBS and incubated with the CD81 + beads (1:6 dilution) overnight at + 4 °C on a plate shaker (500 rpm). The bead-EV complexes were washed with 700 µL/sample of filtered PBS and collected after incubation for 5 min at RT in a magnetic separator by carefully removing the supernatant. The bead-EV complexes were resuspended in 100 µL/sample of filtered PBS and incubated with the following conjugated monoclonal antibodies from Miltenyi Biotec: mouse anti-CD9-APC antibody (Clone REA1071), anti-CD63-FITC antibody (Clone REA1055) and anti-CD81-APC (Clone REA513). 1 µL of antibody was added to each sample (1:100 dilution) and samples were incubated at 4 °C for 1 h on a shaker (500 rpm). Then, 700 µL of filtered PBS were added to each sample and the EV-bead complexes were collected by incubation for 5 min at RT in a magnetic separator; supernatant was removed, and pellets were resuspended in 200 µL of filtered PBS. The samples were analyzed using the ZE5 Cell analyzer (Bio-Rad), with a gate set at 10,000 events for acquisition. Data analysis was performed using Kaluza Analysis 2.1 software.

### Western blot

Western blot analysis for EV specific tetraspanins and Calnexin was performed as previously described [74]. Briefly, equal amounts of protein (1 µg/lane) from fetal derived EVs and fetal chondrocyte’s cell lysate were mixed with Laemmli buffer 4× concentrate (Thermo Scientific, USA, 84788) under nonreducing conditions, denatured for 10 min at 70 °C and loaded onto a precast gel (BioRad, Hercules, USA, 4561093) with 5µL protein marker (Thermo Scientific, USA, 26619). The gel was subjected to electrophoresis (constant 150 V for 1h15min) and electrotransferred onto PVDF membranes (BioRad, Hercules, USA, 1620177). The blotting procedure was carried out in a tank containing an ice block to avoid overheating at constant 370 mA for 1h30min. The membranes were blocked for 1 h in Tris buffered saline with 1mL/L of Tween 20 (Carl Roth, Germany, 27.2) (TBS/T) buffer plus 5% nonfatty milk (Maresi GmbH, Austria) and incubated overnight with primary antibodies; 1:500 rabbit anti-CD9 (Antibodies Online, Germany, ABIN786353), 1:500 rabbit anti-CD81 (Antibodies Online ABIN2789419) and 1:500 goat anti-calnexin (Antibodies Online ABIN1440005) on a shaker at 4 °C. Finally, the membranes were washed 3× in TBS/T and incubated for 1 h with HRP-linked heavy and light chain antibodies 1:1000 HRP goat anti rabbit (Invitrogen, Waltham, MA USA, 32460) or 1:1000 HRP rabbit anti goat (Antibodies Online ABIN964937). The membrane was developed using a Super Signal West Femto protein detection kit (Thermo Scientific, USA, 34094) in an imaging system (ChemiDoc BioRad, Hercules, USA).

### Transmission electron microscopy (TEM)

After tangential flow filtration, 10 µL of ovine fetal-derived EV suspensions (fMSC EV: 1.20E + 11 particles/mL; fCC EV: 1.38E + 11 particles/mL) were incubated on 300 mesh formvar/carbon coated hexagonal copper grids (Science Services, Germany) for 60 min. Samples were washed in ddH20, fixed with 1% glutardialdehyde in Sörensen buffer (pH 7.4) for 5 min, washed again, and then subjected to negative staining with 2% aqueous uranyl acetate for 5 min. Grids were examined using a transmission electron microscope (EM 900, Zeiss, Germany) equipped with a slow-scan CCD 2 K- wide-angle dual-speed camera (TRS, Germany).

The WJ-MSC and P-MSC EVs were imaged by the Electron Microscopy Facility at Vienna BioCenter Core Facilities (VBCF), member of the Vienna BioCenter (VBC), Austria, as follows: for negative staining, carbon-coated 400mesh Cu/Pd grids (Agar Scientific, UK) were glow discharged using a Bal-Tec SCD 005 Suppter Coater (Balzers, Fürstentum Lichtenstein) for 60 s. Four microliters of the sample was then applied to the carbon-coated side of the grid and incubated for 1 min. The grid was blotted, washed two times with 2% phosphotungstic acid or 2% uranyl acetate, and stained with the uranyl acetate or phosphotungstic acid solution for 1 min. Samples were imaged using an FEI Morgagni 268D (FEI, The Netherlands) microscope equipped with a Mega View III CCD (Olympus-SIS) camera.

### Analysis of the therapeutic effect of fetal and perinatal EVs on inflamed ovine adult articular chondrocytes and synoviocytes in 2D and 3D culture

#### Chondrocyte and synoviocyte isolation and culture

The therapeutic efficacy of the four EV treatments was tested on primary ovine adult articular chondrocytes and synoviocytes (*n* = 3 biological replicates), isolated and biobanked from sheep euthanized for reasons unrelated to this study, as previously described [75–77]. Briefly, articular cartilage was minced and digested with collagenase (1 mg/mL collagenase type I, Sigma Aldrich, St. Louis, MO, USA) and filtered through a cell strainer (100 μm, Greiner Bio One, Austria). The obtained chondrocytes were washed twice with PBS +/+ (Mg2+/Ca2+, Gibco, Thermo Fisher Scientific, USA) and centrifuged at 540× g for 5 min at room temperature. The resulting cell pellet was resuspended in standard expansion medium consisting of Dulbecco’s Modified Eagle Medium (Gibco, Thermo Fisher Scientific, USA), 10% Fetal Calf Serum (Capricon Scientific, FBS-12B, Germany), 1% Pen/Strep (100×, Sigma Aldrich, St. Louis, MO, USA) and 1% Amphotericin B (250 µg/mL, Biochrom, M&B Stricker, Germany), and cultured under standard conditions (37 °C, 20% O2, 5% CO2, humidified incubator). Synoviocytes were isolated by enzymatic digestion of the synovial membrane that had been harvested from the femoropatellar and femorotibial joint and incubated with the synovial membrane side facing down in trypsin for 30 min at room temperature. After incubation, the synovial membrane was scraped with a cells scraper and obtained cells were processed analogous to the chondrocytes. All cells were used for therapeutic assays in passage 3.

#### Experimental groups and set-up

The therapeutic efficacy of the four EV treatments was tested on inflamed ovine adult articular chondrocytes and synoviocytes (*n* = 3 biological replicates) cultured in 2D and 3D in StemMACS^®^ medium supplemented with StemMACS^®^ XF and 1% Pen/Strep. To induce inflammation, cells were stimulated with 1ng/mL interleukin-1β (IL-1β) plus 1ng/mL tumor necrosis factor-α (TNF-α) (ImmunoTools, Germany) for 24 h. Subsequently (T0), the cells received fresh medium containing pro-inflammatory cytokines and a dose (1E + 09 particles/mL) of either fCC, fMSC, P-MSCs or WJ-MSCs derived EVs (fCC-EVs, fMSC-EVs, P-MSC-EVs, WJ-MSC-EVs). Healthy, inflamed untreated and dexamethasone (DEX, 40nM) treated inflamed cells served as controls. Chondrocytes and their CM were harvested at 24 h (T24) and 48 h (T48) post treatment for further analyses. Synoviocytes were harvested 48 h (T48) post treatment for further analyses by NGS. Therapeutic efficacy was assessed using viability, proliferation and wound healing assays in 2D culture and next generation mRNA sequencing (NGS) in 3D culture, as previously described [75, 77, 78]. High-resolution mass spectrometry (shotgun proteomics) was performed on the CM of chondrocyte pellet cultures.

All assays were performed with 3 biological replicates per group. For proliferation and EV/cell Interaction and uptake assays, 1000 cells per well were seeded on 96-well plates with six technical replicates per donor. For wound healing (scratch assays) and cell viability (cell metabolic activity, MTT assay), 5000 cells were seeded per well on gelatine-coated 96-well plates with three technical replicates per donor. For 3D culture, chondrocytes (300000 cells) were pelleted in 15mL falcon tubes and synoviocytes (300000 cells) were seeded in Matrigel^®^ (Corning, USA, 354234) and incubated for 72 h before inflammation induction and subsequent experiments.

#### EV/cell interaction and uptake assays

Cellular interaction and uptake of EVs labeled with fluorogenic lipophilic dye AcoDye Aco-600™ (Acoerela, Singapore) was assessed using live-cell imaging, flow cytometry, and confocal microscopy. EVs were labeled with AcoDye Aco-600™ according to the manufacturer’s instructions. Briefly, EVs were diluted in fPBS-/- to a final concentration of 1E + 10 particles/mL and incubated with 1 µM Aco-600™ for 1 h at 37 °C. Excess dye was removed via ultrafiltration at 3000x g for 10 min using Amicon^®^ Ultra filter units (10 kDa cut-off; Merck Millipore, USA), performed three times. The EV pellet was resuspended in StemMACS XF medium and applied to healthy and inflamed chondrocytes.

For EV-cell interaction assays, ovine adult articular chondrocytes (*n* = 3 biological replicates, with six technical replicates per donor per condition) were seeded in 96-well plates (1,000 cells/well). To account for potential interspecies differences in EV uptake, human adult articular chondrocytes (*n* = 1, female, 46 years old) were included as controls. The human chondrocytes, sourced from the ViBiMeD Biobank (Ethics Approval No. 1822/2017), were seeded, cultured, and inflamed following the ovine protocol. Live-cell imaging was conducted using the IncuCyte^®^ S3 Kinetic Imaging System (Sartorius, Germany), capturing images from four fields per well every 30 min over 24 h. The Red Mean Integrated Intensity Object Average per image (RCU µm²/image) was calculated at each time point using the Incucyte software’s (2023 A) Adherent Cell-by-Cell Analysis settings. This method identifies and delineates individual cells within the captured images and measures fluorescence intensity only in regions larger than 500 μm² which contain a nucleus. Controls included untreated cells, labeled EVs without cells, and the dye alone.

For flow cytometry, ovine adult articular chondrocytes (*n* = 3 biological replicates, with two technical replicates per donor per condition) were seeded in 24-well plates (5,000 cells/well). Both healthy and inflamed chondrocytes were treated with the labeled EVs for 1, 6, and 24 h. Following treatment, cells were detached using 0.05% trypsin-EDTA, washed with PBS-−/−(Mg2+/Ca2+), and centrifuged at 540x g for 5 min. The resulting pellets were resuspended in 100 µL PBS–−/−(Mg2+/Ca2+) with DAPI solution (Invitrogen, USA) added immediately prior to flow cytometry. Data were acquired using a FACS Canto II (BD Biosciences, USA) and analyzed with FlowJo 10.8.0 (TreeStar, BD Biosciences). Gates were set to exclude doublets (FSC-W vs. FSC-A) and identify live, DAPI-negative cells. EV uptake was quantified by calculating the percentage of live cells positive for Aco-600™ fluorescence.

For confocal microscopy, representative adult ovine articular chondrocytes (*n* = 1) were seeded in µ-Slide 18 Well polymer-bottom slides (1,000 cells/well; ibidi GmBH, Germany) one technical replicate per condition. Healthy and inflamed cells were treated with labeled EVs for 1 h. Afterwards, cells were washed with warm PBS +/+ (Mg2+/Ca2+) fixed with 4% formalin for 10 min, and permeabilized with 0.2% Triton X-100 for 20 min. Cells were stained with Atto 488 Phalloidin (Sigma-Aldrich, USA) and mounted in VectaShield containing DAPI (ibidi GmBH, Germany). Imaging was conducted using a Nikon Eclipse Ti2-E microscope equipped with a CSU-W1 spinning disk confocal scanner (Yokogawa Electric, Tokyo, Japan) and Photometrics Prime BSI camera (Teledyne Technologies, USA). Z-stacks covering the entire cell volume were captured, and images were processed in FIJI (ImageJ). Identical microscope and acquisition settings were used across experiments. Blue (Dapi), green (Phalloidin) and red (Aco-600™) channels were merged and images were produced using ImageJ (FIJI).

#### Cell viability and proliferation assays

Metabolic activity, an indicator of cell viability, was assessed using the 3-(4,5-dimethylthiazol-2-yl)-2,5-diphenyltetrazolium bromide (MTT) assay (Promega, USA) according to the manufacturer’s instructions. The absorbance was measured with a plate reader (Varioskan LUX, Thermo Scientific, USA) at 595 nm.

For proliferation assays, confluence was determined using the IncuCyte^®^ S3 Kinetic Live cell Imaging System (Sartorius, Germany) which captured pictures at a 2 h interval for 48 h. Cell proliferation rates were calculated based on the increase of confluence levels using the IncuCyte^®^ software (Sartorius, Germany).

#### Wound healing assay

The IncuCyte^®^ WoundMaker tool (Sartorius, Germany) was used to create precise scratches (wounds) in the confluent chondrocyte monolayer. After inflicting the wounds, the cells were washed with PBS Mg2+/Ca2+ (Gibco Life technologies, UK) twice, followed by the addition of medium supplemented with 1ng/mL of interleukin-1 β (IL-1β) and 1ng/mL of tumor necrosis factor-α (TNF-α). The chondrocyte monolayers were imaged every 2 h for a total of 48 h using the IncuCyte^®^ live cell imaging system (Sartorius, Germany) at 10X magnification. For each picture the wound width was calculated using the Scratch Wound analyses pipeline of the IncuCyte^®^ software (Sartorius, Germany). Residual wound sizes were analysed for each timepoint, and wound closure rates were calculated.

#### Next generation sequencing (NGS; mRNA)

For NGS, total RNA was extracted (n = 3 biological replicates, one technical replicate per condition) using the miRNeasy Mini kit (Qiagen, Germany) and the QIAcube (Qiagen, Germany). RNA quality control was performed with the Bioanalyzer RNA 6000 kit (Agilent Technologies, US). Libraries for mRNA (QuantSeq 3’ mRNA-Seq V2 Library Prep Kit with UDI, Lexogen, Austria) small RNA (miRNA, RealSeq Biofluids library preparation kit, RealSeq Biosciences, US) were prepared, pooled in equimolar ratio and sequenced on Illumina NovaSeq SP Flowcell (Illumina Innovative Technologies, USA) in SR100 mode. miND Spike-Ins (TAmiRNA, Austria) were used in the small RNA library preparation for quality control and quantification.

For mRNA analysis, adapter trimmed and quality filtered reads were aligned with STAR v2.7 against the genomic reference Oar-rambouillet.v2.0 provided by Ensembl (https://www.ensembl.org/Ovis_aries_rambouillet/Info/Index)[79], [80]. Assignment of features to the mapped reads was performed with htseq-count v0.13 and differential expression analysis with edgeR v3.30 [81], [82]. A significance cut-off of false discovery rate (FDR)-corrected p-value < 0.05 and a fold change (FC) threshold of |FC| ≥ 1.2 were applied to filter the differentially expressed genes (DEGs). Overlapping and unique DEGs between the different treatment/control groups at 24 h and 48 h were identified using a web-based Venn diagram tool (https://www.interactivenn.net), accessed June 2024). DEGs were further analysed using Ingenuity Pathway Analysis (IPA, Qiagen, USA) to identify enriched pathways, upstream regulators, and potential network connections associated with the identified DEGs.

#### High-resolution mass spectrometry-based proteomics

To identify and quantify proteins in the samples (*n* = 3 biological replicates, one technical replicate per condition), high-resolution mass spectrometry was carried out as previously described [83]. Data analysis including protein identification and label-free quantification (LFQ) was accomplished using MaxQuant software (version 1.6.17.0.22). Raw data were searched against the SwissProt database “homo sapiens” (version 141219, 20380 entries) including an allowed peptide tolerance of 20 ppm, a maximum of two missed cleavages, carbamidomethylation on cysteins as fixed modification as well as methionine oxidation and N-terminal protein acetylation as variable modification. A minimum of one unique peptide per protein was set as search criterium for positive identifications. The “match between runs” option was applied. For all peptide and protein identifications a FDR ≤ 0.01 was set. Using Perseus software (version 1.6.14.0) identified proteins were filtered for reversed sequences and common contaminants. LFQ intensities were transformed (log2(x)), and proteins were additionally filtered for their number of independent identifications (protein identified in 70% of samples in at least one group).

Paired two sample t-tests were carried out for the genuinely secreted proteins identified in EV treated, dexamethasone treated, and healthy samples compared to the inflamed untreated controls. A protein set enrichment analysis (PSEA) was performed using the gene set enrichment analysis (GSEA) software provided by the broad institute (UC San Diego, California, USA) [10.1073/pnas.0506580102]. Proteins were tested against the gene ontology biological process for sheep (Ovis Aries). Prior to PSEA, term - protein associations were filtered for proteins associated with terms representing the extracellular space or extracellular matrix (GO:0010339, GO:0005576, GO:0031012, GO:0043230, GO:0005615) or their offspring. Proteins not associated with any of these terms were removed from the term-protein association database.

### Statistical analysis

Data (except NGS and Mass-Spec data) were analysed using Graphpad Prism (version 10.2.3). Statistical analysis was performed by repeated measures one -way analysis of variance with Geisser-Greenhouse correction and Holm-Sidak’s multiple comparisons tests. P-values < 0.05 were considered statistically significant. Mass-Spec data was analysed using Perseus 1.6.14.0.

## Results

### EV donor cell culture for conditioned medium production

Both WJ-MSC/TERT273 and P-MSC/TERT308 were positive for MSCs markers CD73, CD90 and CD105, and negative for the hematopoietic progenitor cell antigen CD34 (Figure S1).

Both bioreactor-derived and monolayer-cultured fMSCs fulfilled the criteria for defining multipotent mesenchymal stromal cells published by the International Society for Cellular Therapy [84]including adhesion to plastic surfaces, fibroblast-like morphology, expression of MSC surface markers CD44, and CD166, lacking expression of the hematopoietic markers CD31 and CD45 and chondrogenic and osteogenic lineage differentiation potential (Figure S2A-B). Analogous to previous studies using fetal cells, the fMSCs did not show adipogenic differentiation, which may be due to the reduced activation of the proliferator–activated receptor g (PPAR-c) pathway in fetal cells [85–88]. Both bioreactor-derived and monolayer-cultured fCCs exhibited chondrogenic matrix production, as evidenced by the Alcian blue staining of proteoglycans after 21 days in culture (Figure S2C).

### EV isolation and characterization

**Fig. 1:**
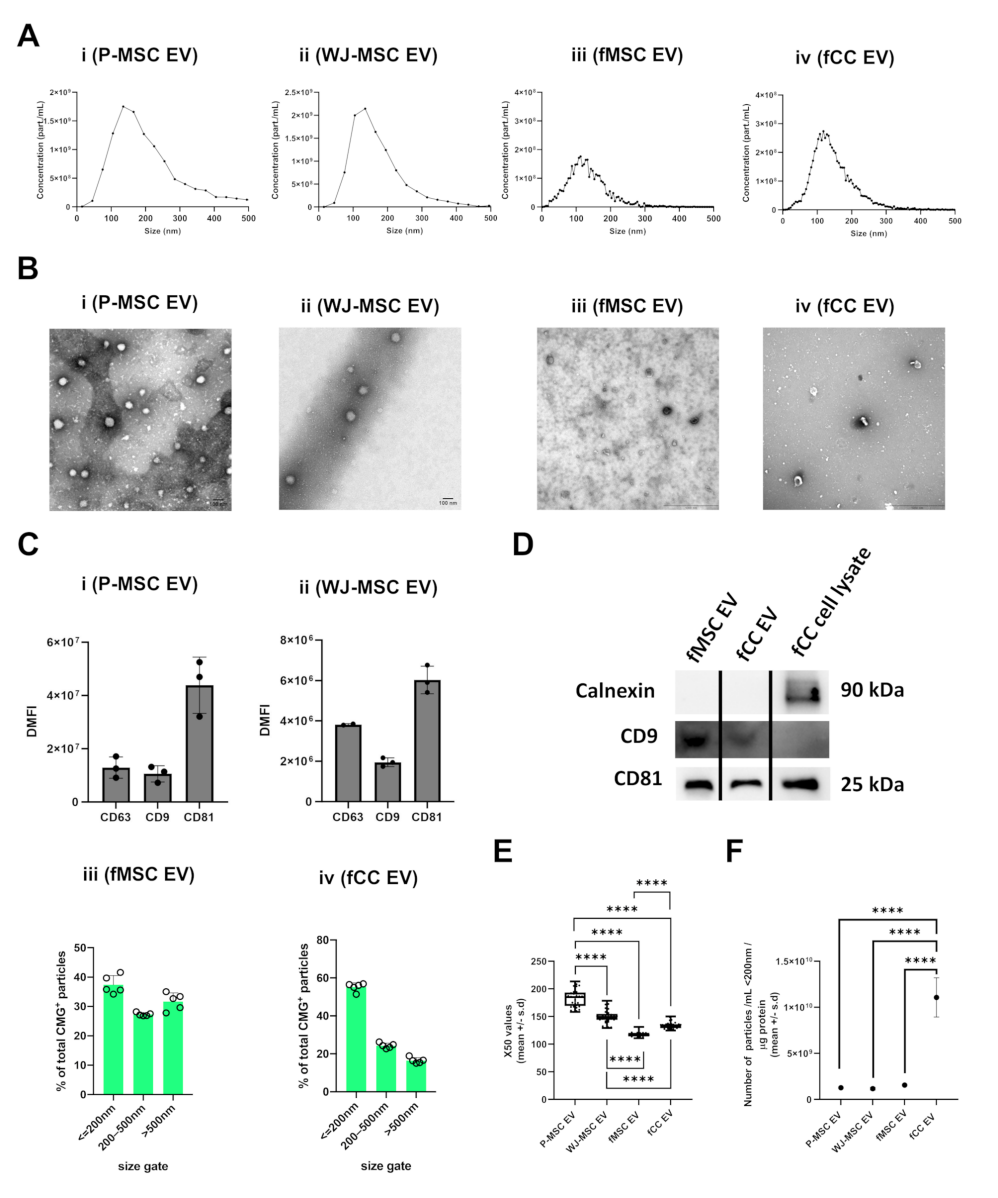
Characterization of ovine fetal and human perinatal derived extracellular vesicles (EV). (**A**) Size distribution curve of (i) P-MSC-EV, (ii) WJ-MSC-EV, (iii) fMSC-EV and (iv) fCC-EV determined by NTA. (**B**) Transelectron Microscopy (TEM) images confirmed the presence of (i) P-MSC-EV, (ii) WJ-MSC-EV, (iii) fMSC-EV and (iv) fCC-EV by showing particles sized 100–500 nm, surrounded by a lipid bilayer with variable electron density cargo. (**C**) Representative image of fluorescence-triggered flow cytometry (FTFC) analysis of i) P-MSC-EV, (ii) WJ-MSC-EV by measuring CD9, CD63, CD81 markers, (iii) fMSC-EV and (iv) fCC-EV by measuring the CMG (CellMask Green) signal separately. (**D**) Western blot analysis of whole-cell lysates and TFF-enriched fetal EVs showed the presence of tetraspanins CD9 and CD81 in both fetal cell-derived EVs, while calnexin, a non-EVmarker, was only detected in the cell lysate. For representation purposes, images were cropped around the designated molecular weight of each protein of interest and compiled into a single panel. Full-length, uncropped blots are provided in Figure S3. (**E**) Box plots (mean ± error) showing individual X50 values (nm) determined by NTA of all 4 EVs. (**F**) Scatter plots (mean ± error) showing EVs < 200 nm normalized by total protein concentration. Not significant (ns, *p* ≥ 0.05), *p < 0.05, **p < 0.01, ***p < 0.001

Nanoparticles were successfully isolated from all 4 donor cell types, each exhibiting characteristics indicative of EVs. These characteristics included presence of particles < 200 nm, as determined by NTA, FT-FC and TEM (Fig. 1A-D). Transmission electron microscopy further confirmed the presence of EVs, showing particles sized 100–500 nm, with variable electron density cargo (Fig. 1B). The presence of a lipid bilayer membrane (FT-FC) and expression of tetraspanins (CD9, CD63, CD81), as determined by beads-based flow cytometry for perinatal-derived EVs (Fig. 1C) and Western-Blot (CD9 and CD81) for fetal cell EVs (Fig. 1D, Figure S3). In fetal chondrocytes, the non-EV marker calnexin was highly expressed on a cellular level, while EVs showed no expression (Fig. 1D).

The four different EV donor cell types yielded different numbers of particles/ml with fCCs and fMSCs producing approximately half as many particles/ml as P-MSCs and about 1/3 less than WJ-MSC-EVs (Table 1), however the differences did not reach statistical significance (*p* = 0.6799). P-MSC EV had significantly higher (*p* < 0.0001) median particle size (X50 values) than any other EV type, WJ-MSC EV had significantly higher (*p* < 0.0001) median particle size (X50 values) compared to both fetal derived EVs and fMSC EV had significantly lower (*p* < 0.0001) median size particles than fCC EV (Fig. 1E).

**Table 1:** Characterization of ovine fetal and human perinatal derived extracellular vesicles (EV). Characterization of EV isolates from primary ovine fetal umbilical cord blood derived MSCs (fMSCs), primary ovine fetal articular chondrocytes (fCC), immortalized human perinatal wharton’s jelly derived MSCs (WJ-MSCs, cell line: TERT273) or immortalized human perinatal Amnion derived MSCs (P-MSCs, cell line: TERT308) by particle size distribution and number by nanoparticle tracking analysis (NTA)and total protein concentration measurement by qubit. For the primary cells, fCCs and fMSCs, measurements are provided as mean of the biological replicates (by donor) and as the characteristics of the treatment pool derived from the biological replicates (pool)

Cell type	Total Particles/ mL	Particles < 200 nm / mL	Percent particles < 200 nm (%)	X50 (nm)	Number of Traces	Average Number of Particles per frame	Protein conc. (µg/µL)	EVs < 200 nm/ µg protein
	Mean (+/- s.d)	Mean (+/- s.d)		Mean (+/- s.d)	Mean (+/- s.d)	Mean (+/- s.d)	Mean (+/- s.d)	Mean (+/- s.d)
fCC-EVs by donor	1.68E + 11 (1.85E + 11)	1.28E + 11 (1.41E + 11)	76.28	161 (18)	161 (123)	110 (92)	3.17 (0.66)	3.94E + 10 (3.9E + 10)
fCC-EVs pool	5.44E + 10 (7.82E + 09)	3.94E + 10 (6.01E + 09)	72.41	134.85 (7.42)	398 (119)	375 (126)	3.56 (0.01)	1.11E + 10 (1.74E + 09)
fMSC-EVs by donor	5.38E + 10 (5.15E + 10)	4.52E + 10 (4.44E + 10)	84.08	162 (10)	243 (55)	184 (56)	2.91 (0.46)	1.89E + 10 (2E + 10)
fMSC-EVs pool	6.42E + 09 (2.65E + 08)	4.76E + 09 (2.45E + 08)	74.23	117.34 (5.03)	620 (164)	460 (24)	3.06 (0.01)	1.55E + 09 (7.02E + 07)
P-MSC-EVs	1.12E + 10 (9.83E + 07)	4.501E + 09 (1.55E + 08)	59.88	182.50 (15.23)	149.0 (23.13)	105 (18)	5.3	1.27E + 09 (1.47E + 07)
WJ-MSC-EVs	1.02E + 10 (8.55E + 08)	7.68E + 09 (6.0E + 08)	77.07	149.68 (11.57)	77.45 (17.26)	56 (12)	6.74	1.17E + 09 (9.64E + 07)
fPBS	0E + 00	0E + 00	-	-	7 (13)	5 (11)	-	-

Total protein concentration varied significantly (fMSC EV versus P-MSC EV: *p* = 0.02269; P-MSC EV versus WJ-MSC EV: *p* = 0.0244, Table 1). fCC EV had significantly more EVs < 200 nm/µg protein compared to any other EV type (*p* < 0.0001, Fig. 1F).

The average number of particles per frame ranged between 110 and 460 particles (Table 1). In the buffer control (fPBS -/-) no particles were detected.

### EV cell interaction and uptake

**Fig. 2:**
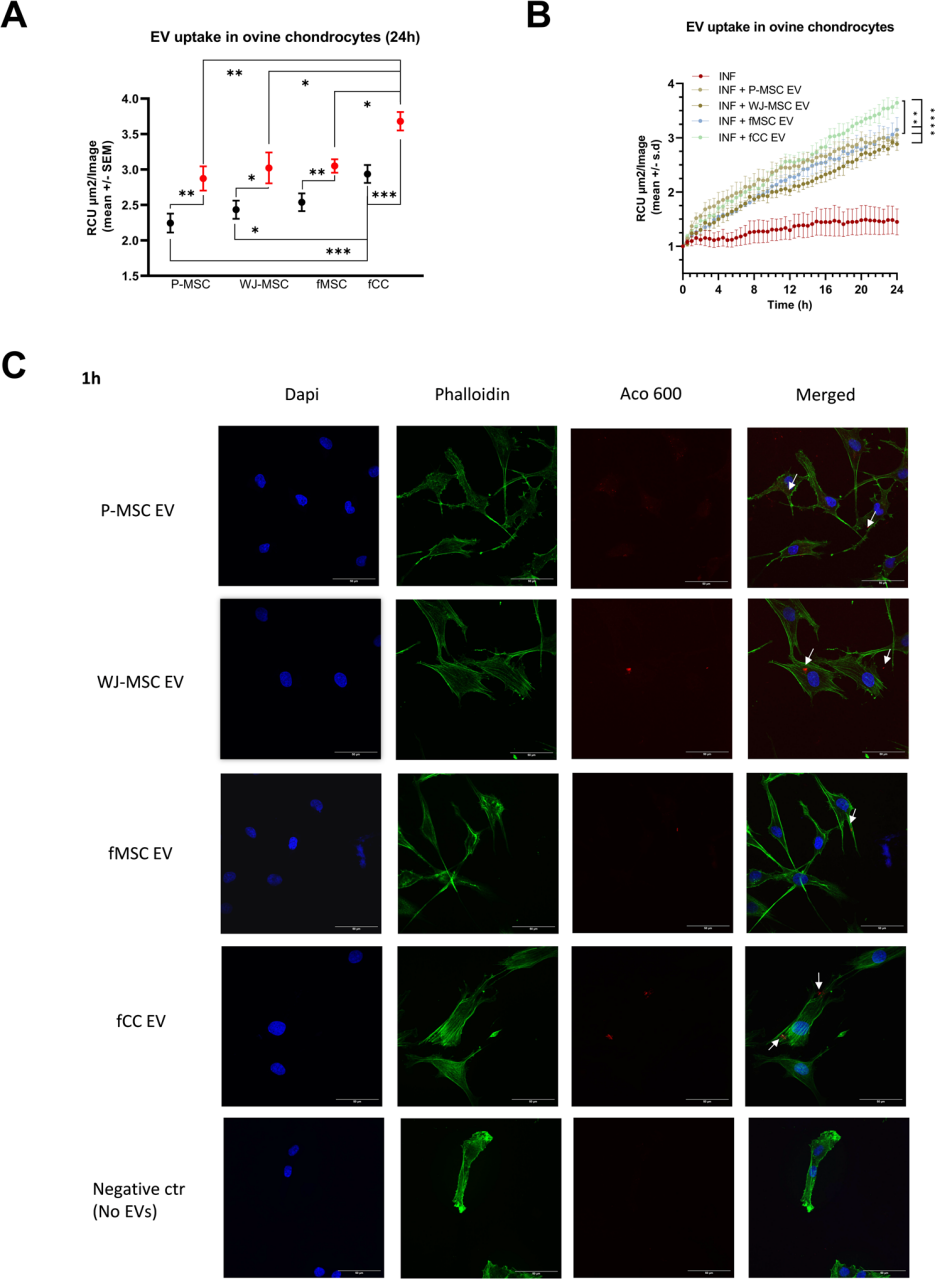
EV-cell Interaction: Live cell imaging assay. (**A**) Cellular red fluorescent signal intensity (Red Mean Intensity Object Average (RCU, mean ± standard error of the mean (SEM)) over time as an indicator of EV uptake efficiency in healthy (black symbols ) and inflamed (red symbols) chondrocytes. All four EV types successfully interacted with both inflamed and healthy chondrocytes within a 24-hour period. EV type significantly affected EV incorporation (F = 15.80. DFn = 3, DFd = 78, *p* > 0.0001) with fCC EVs having a significantly higher affinity compared to all other EV treatments in both healthy and inflamed conditions (*p* < 0.0001 compared to fMSC-EVs and P-MSC-EVs, *p* = 0.0005 compared to WJ-MSC-EVs). Inflamed cells exhibited a higher uptake of EVs across all treatments compared to healthy cells (F = 41.46. DFn = 1, DFd = 78, *p* < 0.0001). Ovine adult articular chondrocytes (*n* = 3 biological replicates) with six technical replicates per donor per condition. (**B**) Over 24 h, red fluorescent signal intensity significantly increased for all treatments (*p* < 0.0001), with fCC-EVs demonstrating the steepest increase in inflamed ovine adult articular chondrocytes (fMSC-EVs: *p* < 0.0001; P-MSC-EVs: *p* = 0.0270; WJ-MSC-EVs: *p* < 0.0001). Ovine adult articular chondrocytes (*n* = 3 biological replicates) with six technical replicates per donor per condition. (**C**) Representative confocal microscopy images illustrating visually but not quantitatively the uptake of (i) P-MSC-EV, (ii) WJ-MSC-EV, (iii) fMSC-EV and (iv) fCC-EV by inflamed adult ovine chondrocytes. Ovine adult articular chondrocytes (*n* = 1 biological replicate) with one technical replicate per condition. Not significant (ns, *p* ≥ 0.05), *p < 0.05, **p < 0.01, ***p < 0.001

All four EV types interacted successfully with both inflamed and healthy chondrocytes, as evidenced by cellular red fluorescent signal intensity (RCU) and flow cytometry (Fig. 2A-B, Figure S4). Confocal microscopy confirmed EV uptake in inflamed ovine adult articular chondrocytes, revealing a diffuse localization of labeled EVs within the cytoplasm and 1-hour post-treatment (Fig. 2C).

Over 24 h, red fluorescent signal intensity significantly increased for all treatments (*p* < 0.0001), with fCC-EVs demonstrating the steepest increase in inflamed ovine adult articular chondrocytes (fMSC-EVs: *p* < 0.0001; P-MSC-EVs: *p* = 0.0270; WJ-MSC-EVs: *p* < 0.0001; Fig. 2B). No signal was detected in the wells containing stained EVs without cells, and a consistent, unchanged signal was observed in cells incubated with EVs alone (Fig. S4C).

EV source significantly influenced EV incorporation (*p* < 0.0001, F = 27.5, DFn = 4, DFd = 8) with fCC EVs achieving the highest uptake across conditions. In inflamed ovine adult articular chondrocytes, fCC-EVs resulted in significantly greater red fluorescence at 24 h compared to all other treatments (fMSC-EVs: *p* = 0.0285; P-MSC-EVs: *p* = 0.0033; WJ-MSC-EVs: *p* = 0.0214; Fig. 2A). In healthy cells, fCC-EVs resulted in significantly greater red fluorescence at 24 h compared to perinatal EVs (P-MSC-EVs *p* = 0.0010, WJ-MSC-EVs *p* = 0.0225, Fig. 2A). Inflamed cells generally exhibited higher EV uptake than healthy cells across all EV types (*p* = 0.0033; F = 300.5; DFn = 1; DFd = 2).

Flow cytometry corroborated these findings, showing a time-dependent increase in the percentage of positive fluorescent cells over the 24-hour period (*p* < 0.0001; F = 53.53; DFn = 2; DFd = 48), with fCC-EVs again achieving the highest levels (Figure S4D-E).

All four EV types were also successfully taken up by inflamed human adult articular chondrocytes (Figure S4A-B). Notably, the ovine fetal-derived EVs (fCC-EVs and fMSC-EVs) outperformed human perinatal EVs in uptake efficiency in human cells. Specifically, fCC-EVs achieved significantly higher cellular red fluorescent signal intensity than WJ-MSC-EVs (*p* = 0.0007) and P-MSC-EVs (*p* = 0.0005) and non-significantly higher than fMSC-EVs (*p* = 0.3845). FMSC EVs were also taken up more efficiently than WJ-MSC-EVs (*p* = 0.0098) and P-MSC-EVs (*p* = 0.0078; Figure S4A). These findings confirmed that the superior uptake efficiency of fetal EVs, especially fCC EVs, was independent of recipient cell species.

#### Therapeutic efficacy of EVs - cell viability, proliferation and wound healing assays

Treatment with P-MSC-EVs significantly increased (*p* = 0.0014) viability of inflamed ovine adult articular chondrocytes compared to untreated controls at T48 (Figure S5B). Both fetal-derived EVs significantly enhanced (fMSC-EVs: *p* = 0.0065; fCC-EVs: *p* = 0.0232) the viability of inflamed ovine adult synoviocytes compared to untreated inflamed cells at T48 (Figure S5D). However, neither fetal nor perinatal-derived EVs significantly affected cell proliferation or scratch wound closure in inflamed ovine adult articular chondrocytes or synoviocytes (Figure S5E-H).

### Therapeutic efficacy of EVs - Next generation sequencing (mRNA)

**Fig. 3:**
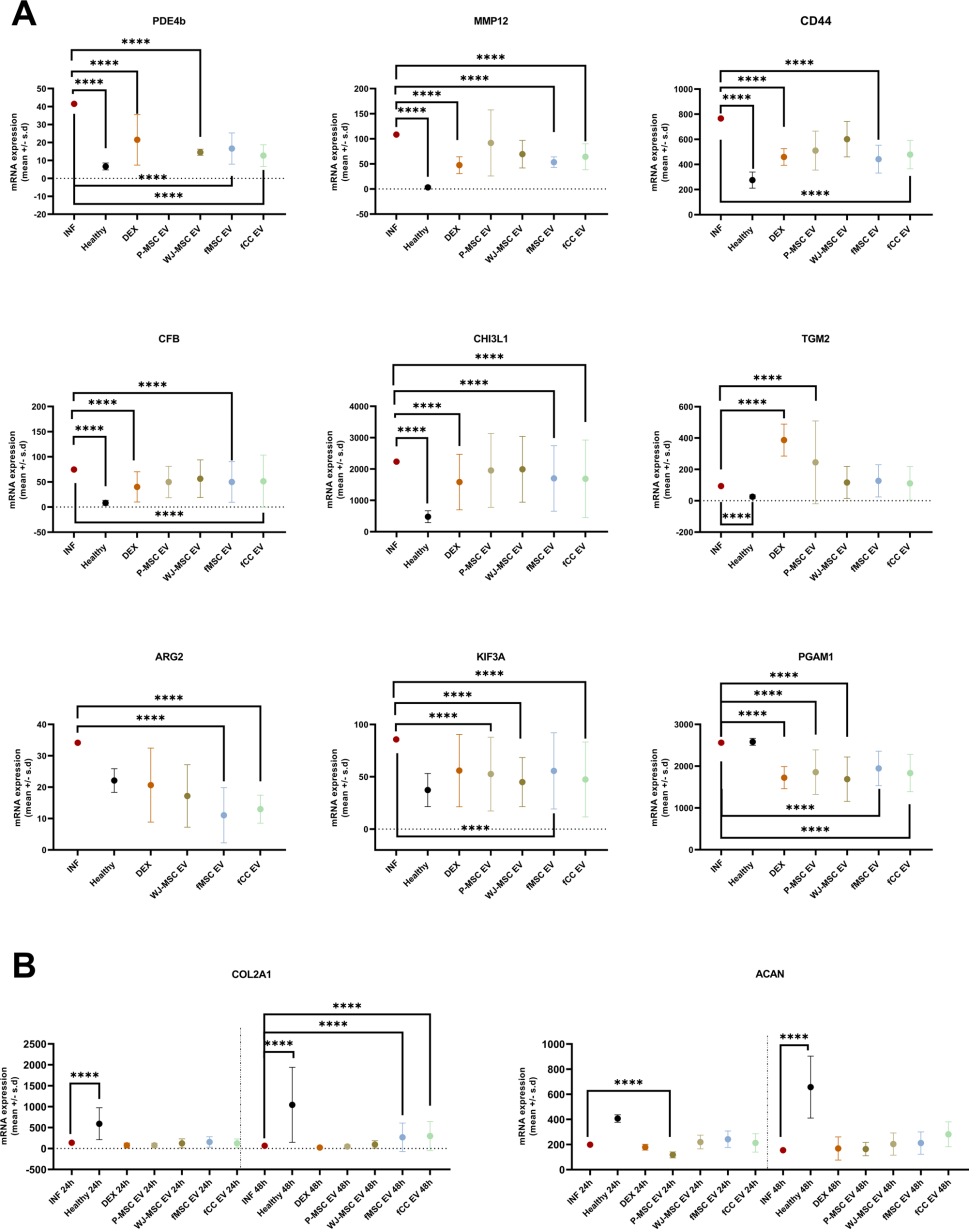
Treatment effect of four different extracellular vesicles (EVs) on inflamed chondrocytes at 24 h (mRNA analysis). (**A**) Scatter plots (mean ± SD) showing inflammation related genes were significantly downregulated in treated inflamed chondrocytes compared to untreated inflamed chondrocytes, including Phosphodiesterase 4B (PDE4B), Matrix Metalloproteinase 12 (MMP-12), CD44, complement Factor B (CFB), chitinase-3 like-protein-1 (CHI3L1), kinesin family number 3 A (KIF3A), Arginase 2 (ARG 2) and Phosphoglucomutase-1 (PGAM1). Transglutaminase 2 (TGM2) was significantly upregulated with DEX and P-MSC EV treatment compared to untreated inflamed chondrocytes. (**B**) Scatter plots (mean ± SD) showing Extracellular matrix (ECM)-related gene Col2a1 was significantly upregulated in fMSC-EV and fCC-EV treated inflamed cells but ACAN was significantly downregulated in P-MSC-EV treated chondrocytes. Ovine adult articular chondrocytes (*n* = 3 biological replicates) with one technical replicate per donor per condition. Not significant (ns, *p* ≥ 0.05), *p < 0.05, **p < 0.01, ***p < 0.001

All four EV treatments significantly reduced the inflammatory response in inflamed ovine adult articular chondrocytes at T24 compared to untreated inflamed controls, similar to the anti-inflammatory glucocorticoid DEX. This was indicated by downregulation of selective genes associated with OA inflammation pathways, including Matrix Metalloproteinase 12 (MMP-12), Chitinase 3 like 1 (CH3L1), Phosphodiesterase 4B (PDE4B) and complement factor B (CFB, Fig. 3A). The therapeutic effect was also evident in the overall number of DEGs in the comparison of EV-treated versus untreated inflamed cells, with fCC-EVs achieving the greatest difference to inflamed cells (89 DEGs) and P-MSC-EVs the lowest (57 DEGS) (Tables 2 and 3, Table S1-S4, Figure S6 and S7). Although the number of DEGs between healthy and inflamed chondrocytes increased from T24 (56 DEGs) to T48 (111 DEGs), the number of DEGs between all treated (EVs and DEX) and untreated inflamed chondrocytes decreased, indicating diminishing anti-inflammatory efficacy (Tables 2 and 3 and Table S1 and S3, Figure S6 and S7). In contrast, the effects on cartilage extracellular matrix (ECM) genes became more apparent at T48 (Fig. 3B, Tables S3 and S4). Only fCC-EVs and fMSC-EVs significantly increased expression of collagen 2a (COL2a) in inflamed chondrocytes at T48, bringing it closer to healthy control levels (Fig. 3B, Tables S3 and S4). In contrast, P-MSC-EVs significantly downregulated aggrecan (ACAN) at T24, while other EV treatments non-significantly increased ACAN expression (Fig. 3B, Table S1 and S2).

**Table 2:** Number of differentially expressed genes (DEGs) between the treatments/control groups and untreated inflamed (infl.). Number of differentially expressed genes (DEGs) between the treatment/control groups and untreated inflamed (infl.) chondrocytes and infl. Synoviocytes. Cells were treated with extracellular vesicles (EVs) derived from fetal ovine chondrocytes (fCC-EVs), fetal ovine umbilical cord blood MSCs (fMSCs-EVs), immortalized human perinatal wharton’s jelly derived MSCs (WJ-MSCs, cell line: TERT273) or immortalized human perinatal Amnion derived MSCs (P-MSCs, cell line: TERT308) or dexamethasone (DEX). Chondrocytes were harvested 24 h (T24) and 48 h (T48) after treatment, synoviocytes at T48 for next generation sequencing

Cell type	Treatment/control group comparison	Time	Overall DEGs	Up - regulated DEGs	Down - regulated DEGs
Chondrocytes	Healthy vs. infl.	T24	56	13	43
		T48	111	55	56
	DEX vs. infl.	T24	168	69	99
		T48	5	4	1
	fCC-EVs vs. infl.	T24	89	9	80
		T48	1	1	0
	fMSC-EVs vs. infl.	T24	85	9	76
		T48	2	1	1
	PMSC-EVs vs. infl.	T24	57	23	34
		T48	7	4	3
	WJ-MSC-EVs vs. infl.	T24	69	9	60
		T48	1	0	1
Synoviocytes	Healthy vs. infl.	T48	133	46	87
	DEX vs. infl.	T48	109	67	42
	fCC-EVs vs. infl.	T48	43	18	25
	fMSC-EVs vs. infl.	T48	98	28	70
	PMSC-EVs vs. infl.	T48	16	11	5
	WJMSC-EVs vs. infl.	T48	7	4	3

**Table 3:**
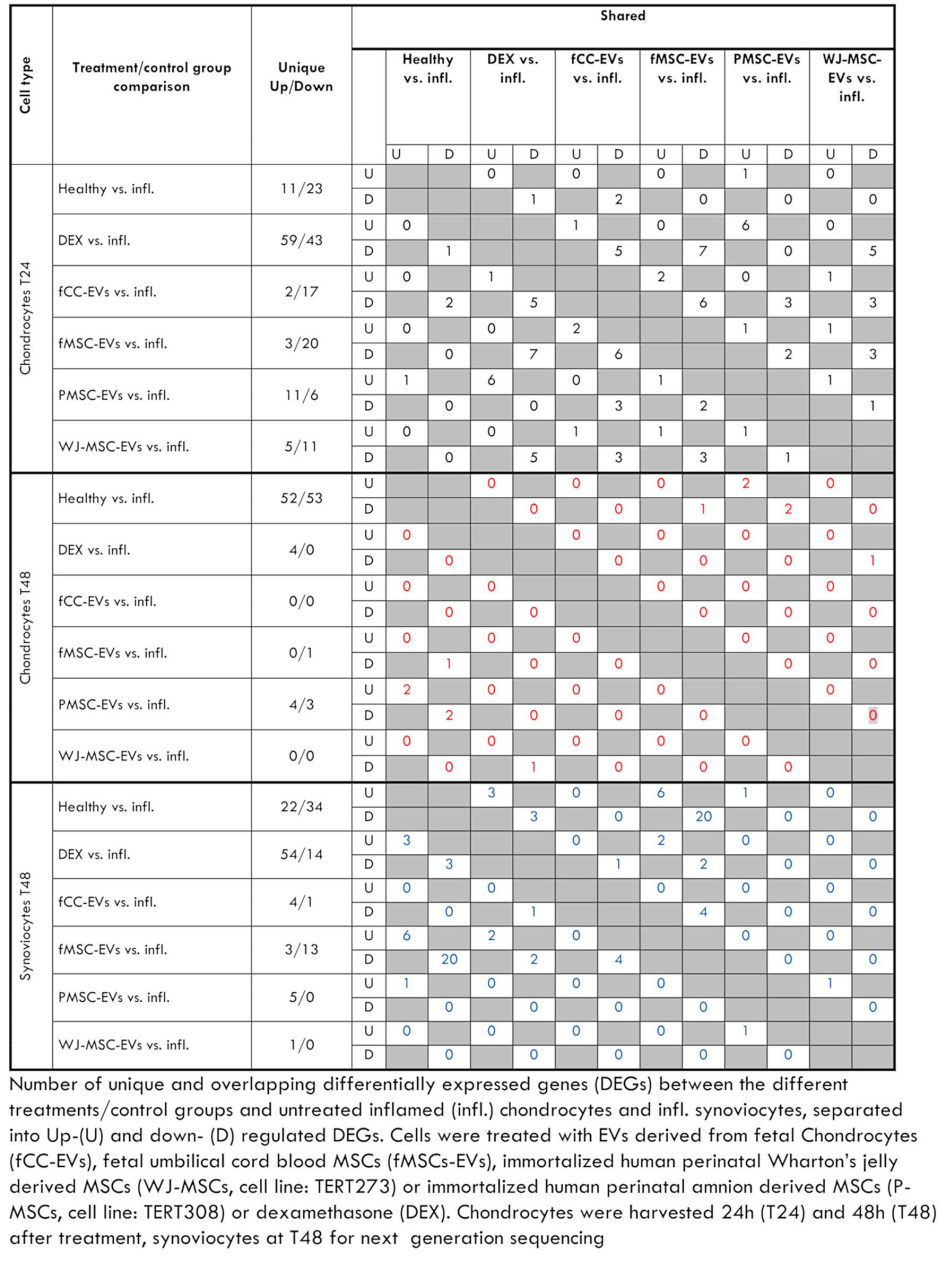
Unique and overlapping differentially expressed genes (DEGs) between the treatments/control groups and untreated inflamed (infl.)

Venn analysis of DEGs identified unique and overlapping DEGS contrasting healthy versus inflamed chondrocytes and those treated with various EVs and DEX at T24 and T48 (Table S1 and S3, Figure S6 and S7) as well as for overlapping or unique DEGs in ovine adult articular chondrocytes between different EV treatments 24 h and 48 h after treatment (Table S2 and S4).

**Fig. 4:**
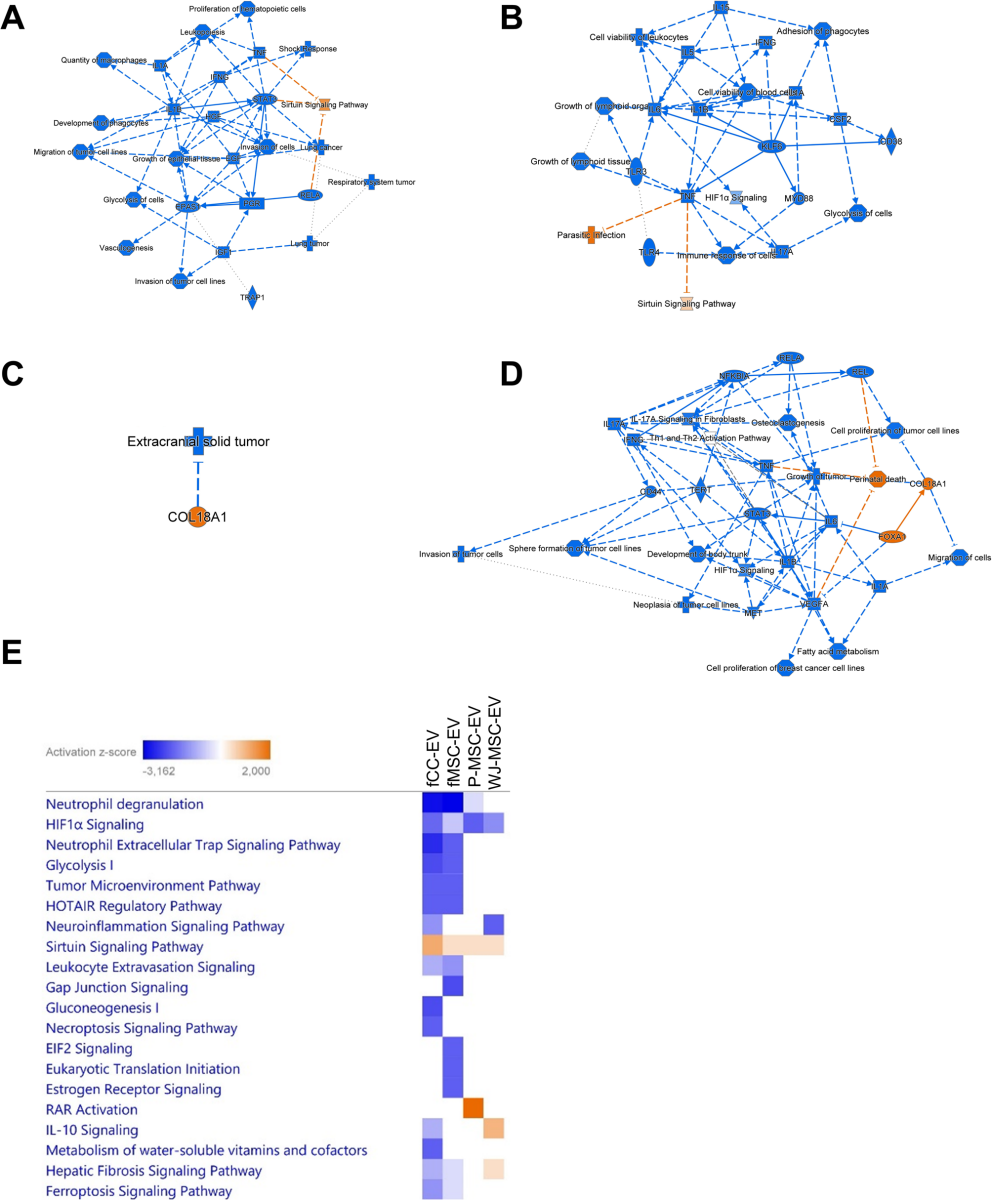
Treatment effect of four different extracellular vesicles (EVs) on inflamed chondrocytes at 24 h (Pathway analysis). Analysis of the treatment effect of EVs harvested from ovine fetal articular chondrocytes (fCC-EVs), ovine fetal umbilical cord blood derived MSCs (fMSC-EVs, human perinatal placental amnion (P-MSC-EVs) derived MSCs or human perinatal Wharton’s Jelly (WJ-MSC-EVs) derived MSCs on inflamed chondrocytes 24 h after treatment was carried out using Ingenuity Pathway Analysis. (**A**) Graphical summary of the core analysis for genes differentially expressed (DEGs) between fCC-EV-treated and untreated inflamed chondrocytes. The graphical summary selects and connects a subset of the most significant entities predicted in the analysis, including canonical pathways, upstream regulators, diseases and biological functions, to visualize related biological activities. (**B**) Graphical summary of the core analysis for DEGs between fMSC-EV-treated and untreated inflamed chondrocytes. (**C**) Graphical summary of the core analysis for DEGs between P-MSC-EV-treated and untreated inflamed chondrocytes. (**D**) Graphical summary of the core analysis for DEGs between WJ-MSC-EV-treated and untreated inflamed chondrocytes. (**E**) Top twenty canonical pathways significantly regulated 24 h following treatment of inflamed chondrocytes with the four EVs types. The effects of DEGs on pathway activation or repression were determined using activation *z* scores. The intensity of a block colour corresponds to down-regulated (blue) and up-regulated (orange). All significant top 10 pathways for each treatment group are listed in Table S6. Individual differential expressed genes (DEGs) contributing to the activation or inhibition of canonical pathways identified by Ingenuity Pathway Analysis in ovine adult articular chondrocytes at 24 h after treatment with EVs harvested ovine fetal articular chondrocytes (fCC-EV), ovine fetal umbilical cord blood derived MSCs (fMSC-EVs), human perinatal Wharton’s Jelly (WJ-MSC-Evs) or amnion (P-MSC-EVs) derived MSCs compared to untreated inflamed chondrocytes are listed in Tables S9-12

Pathway analysis comparing the canonical pathways between the four conditions (fCC-EVs vs. INF, fMSC-EVs vs. INF, P-MSC-EVs vs. INF, WJ-MSC-EVs vs. INF) at T24 show distinct biological activity of the different EV sources (Fig. 4A-E, Table S4). All 4 EVs inhibited the “HIF1A signaling” and activated the “sirtuin signaling” pathway to varying extents, with fCC-EVs leading to the highest activation of “sirtuin signaling”. Treatment with the two perinatal EVs yielded no pathways shared uniquely between P-MSC-EVs and WJ-MSC-EVs treated chondrocytes. Fetal EV treatment resulted in a total of 12 shared pathways, 5 of which were shared exclusively with each other, namely downregulation of the “neutrophil extracellular trap signalling”, “HOTAIR regulatory”, “tumour microenvironment”, “ferroptosis signalling” and “leukocyte extravasation signalling” pathways, showing a clear clustering of fetal versus perinatal EVs in the enriched pathways (Fig. 4A-E, Table S7, for individual molecules responsible for the activation or inhibition of specific pathways see Tables S9-S9).

**Fig. 5:**
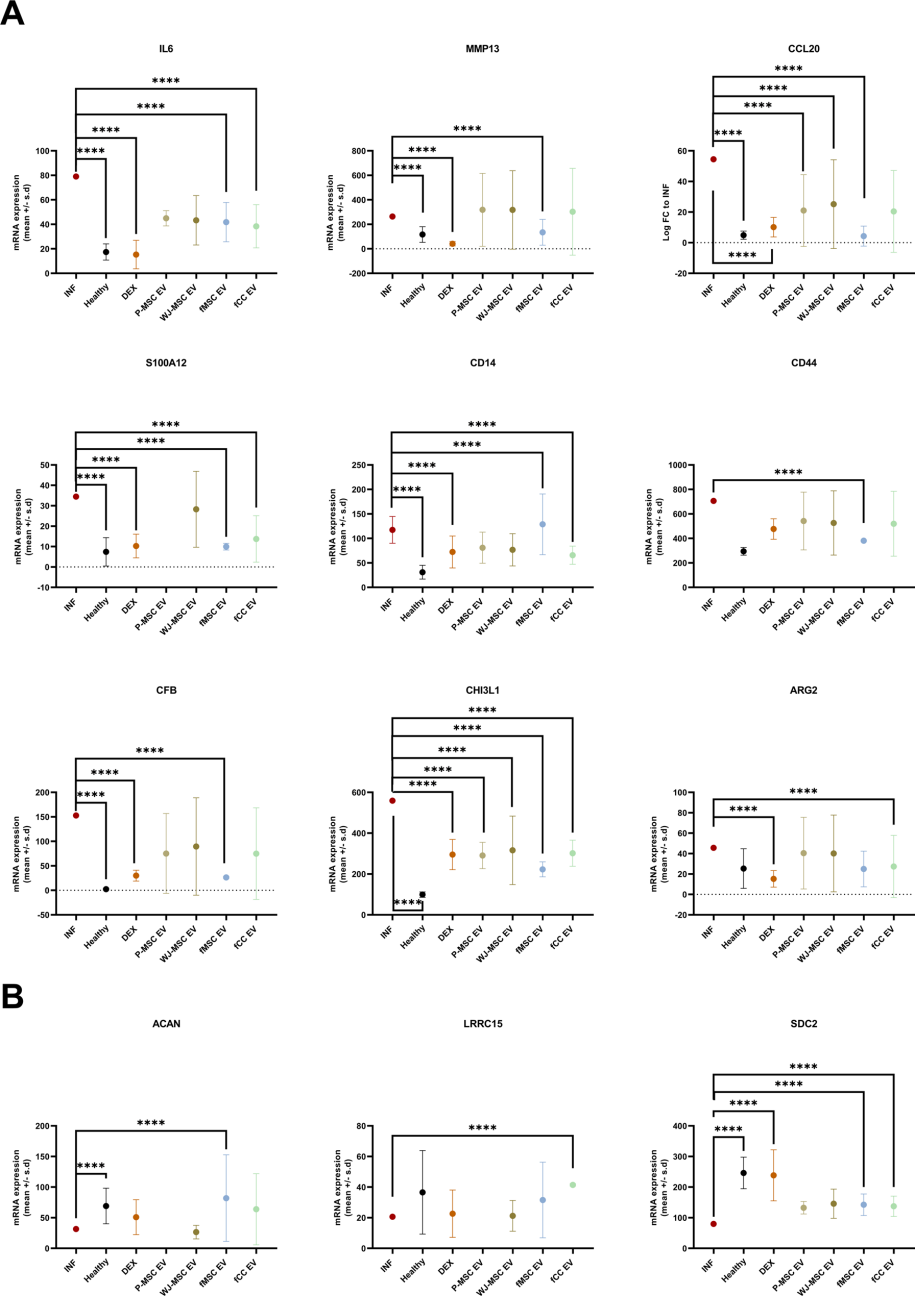
Treatment effect of four different extracellular vesicles (EVs) on inflamed synoviocytes at 48 h (mRNA analysis). (**A**) Scatter plots (mean ± SD) showing inflammation related genes were significantly downregulated in treated inflamed adult synoviocytes compared to untreated inflamed synoviocytes, including Interleukin 6 (IL-6), Matrix Metallopeptidase 13 (MMP13), CC-chemokine ligand 20 (CCL20), S100 calcium-binding protein A12 (S100A12), CD14, CD44, complement factor (CFB), chitinase 3 like protein 1 (CHI3L1) and Arginase 2 (ARG 2). (**B**) Scatter plots (mean ± SD) showing Extracellular matrix (ECM)-related gene ACAN was significantly only upregulated with fMSC EV, Leucine rich repeat containing 15 (LRRC15) was only significantly upregulated with fCC EV and Syndecan 2 (SDC2) was significantly upregulated with DEX and with both fetal derived EVs. Ovine adult synoviocytes (*n* = 3 biological replicates) with one technical replicate per donor per condition. Not significant (ns, *p* ≥ 0.05), *p < 0.05, **p < 0.01, ***p < 0.001

In inflamed ovine synoviocytes, analogous to chondrocytes, both fetal and perinatal cell-derived EV treatments significantly reduced the inflammatory response at T48 compared to inflamed untreated controls. This was indicated by the significant downregulation of selective genes associated with OA inflammation pathways, including interleukin-6 (IL6), S100 calcium-binding protein A12 (S100A12) and CD14 in synoviocytes treated with fCC-EVs, fMSC-EVs or DEX, CFB and MMP13 after fMSC-EVs and DEX and CHI3L1 after all treatments (Fig. 5). The therapeutic effect was also reflected in the number of DEGs in the comparison of EV-treated versus untreated inflamed cells, with fMSC-EVs achieving the greatest difference to inflamed cells (98 DEGs) and WJ-MSC-EVs the least (7 DEGS) (Tables 2 and 3, Tables S5 and S6, Figure S8). Additionally, the anti-fibrotic proteoglycan syndecan-2 (SDC2) was significantly upregulated in fCC-EVs, fMSC-EVs or DEX treated synoviocytes and ACAN in fMSC-EV treated cells (Fig. 5B).

Venn analysis of DEG in synoviocytes identified unique and overlapping DEGS contrasting healthy versus inflamed synoviocytes and those treated with various EVs and DEX (Table S5, Figure S8). Synoviocytes treated with fMSC-EVs shared most DEGs (26, 6 up- and 20 down-regulated) with healthy controls, followed by P-MSC EV (1 up-regulated), those treated with fCC-EVs and WJ-MSC-EVs none. Fetal EV-treated (fCC-EVs and fMSC-EVs) synoviocytes shared 4 DEGs (4 down-regulated), perinatal EV-treated (P-MSC-EVs and WJ-MSC-EVs) shared 1 DEGs (1 up-regulated) and all 4 EV treatments did not share any DEGs (Table 3). Overlapping or unique DEGs in ovine adult synoviocytes were also identified between different EV treatments 48 h after treatment (Table S6).

**Fig. 6:**
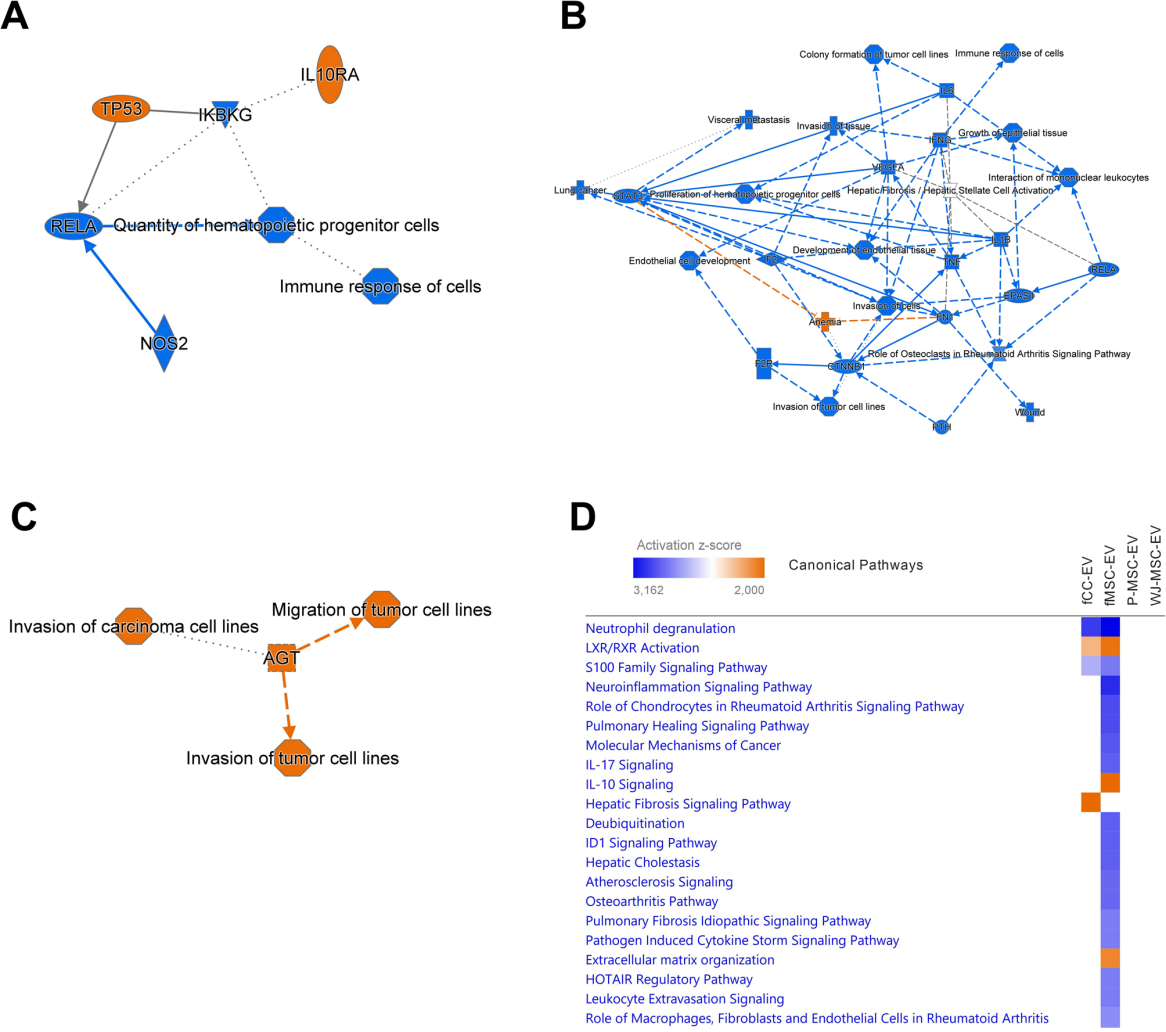
Treatment effect of four different extracellular vesicles (EVs) on inflamed synoviocytes at 48 h (Pathway analysis). Analysis of the treatment effect of EVs harvested from ovine fetal articular chondrocytes (fCC-EVs), ovine fetal umbilical cord blood derived MSCs (fMSC-EVs, human perinatal placental amnion (P-MSC-EVs) derived MSCs or human perinatal Wharton’s Jelly (WJ-MSC-EVs) derived MSCs on inflamed synoviocytes 48 h after treatment was carried out using Ingenuity Pathway Analysis. (**A**) Graphical summary of the core analysis for genes differentially expressed (DEGs) between fCC-EV-treated and untreated inflamed synoviocytes. The graphical summary selects and connects a subset of the most significant entities predicted in the analysis, including canonical pathways, upstream regulators, diseases and biological functions, to visualize related biological activities. (**B**) Graphical summary of the core analysis for DEGs between fMSC-EV-treated and untreated inflamed synoviocytes. (**C**) Graphical summary of the core analysis for DEGs between P-MSC-EV-treated and untreated inflamed synoviocytes. (**D**) Graphical summary of the core analysis for DEGs between WJ-MSC-EV-treated and untreated inflamed synoviocytes. (**E**) Top twenty canonical pathways significantly regulated 24 h following treatment of inflamed synoviocytes with the four EVs types. The effects of DEGs on pathway activation or repression were determined using activation *z* scores. The intensity of a block colour corresponds to down-regulated (blue) and up-regulated (orange). All significant top 10 pathways for each treatment group are listed in Table S7. Individual differential expressed genes (DEGs) contributing to the activation or inhibition of canonical pathways identified by Ingenuity Pathway Analysis in ovine adult articular chondrocytes at 24 h after treatment with EVs harvested ovine fetal articular chondrocytes (fCC-EV), ovine fetal umbilical cord blood derived MSCs (fMSC-EVs), human perinatal Wharton’s Jelly (WJ-MSC-Evs) or amnion (P-MSC-EVs) derived MSCs compared to untreated inflamed chondrocytes are listed in Tables S13-16

Pathway analysis comparing the canonical pathways between the four conditions (fCC-EVs vs. INF, fMSC-EVs vs. INF, P-MSC-EVs vs. INF, WJ-MSC-EVs vs. INF) at T48 revealed that the two fetal EV preparations inhibit the “neutrophil degranulation” and “S100 family signaling” pathways and activate the “LXR/RXR” pathway (Fig. 6A-D, Table S8). fMSC-EVs treated cells dominated the comparative canonical pathway list with inhibition of various proinflammatory pathways such as the “role of chondrocytes in rheumatoid arthritis” and “osteoarthritis” pathways and activation of the “IL-10” and “extracellular matrix organization” pathways (Fig. 6, Table S8, for individual molecules responsible for the activation or inhibition of specific pathways see Tables S13-S16).

#### Therapeutic efficacy of EVs –Proteomics

High-resolution mass spectrometry identified a total of 395 proteins in the chondrocyte secretome (FDR < 0.01) of which 85 proteins were confirmed to be genuinely secreted 48 h post treatment.

**Fig. 7:**
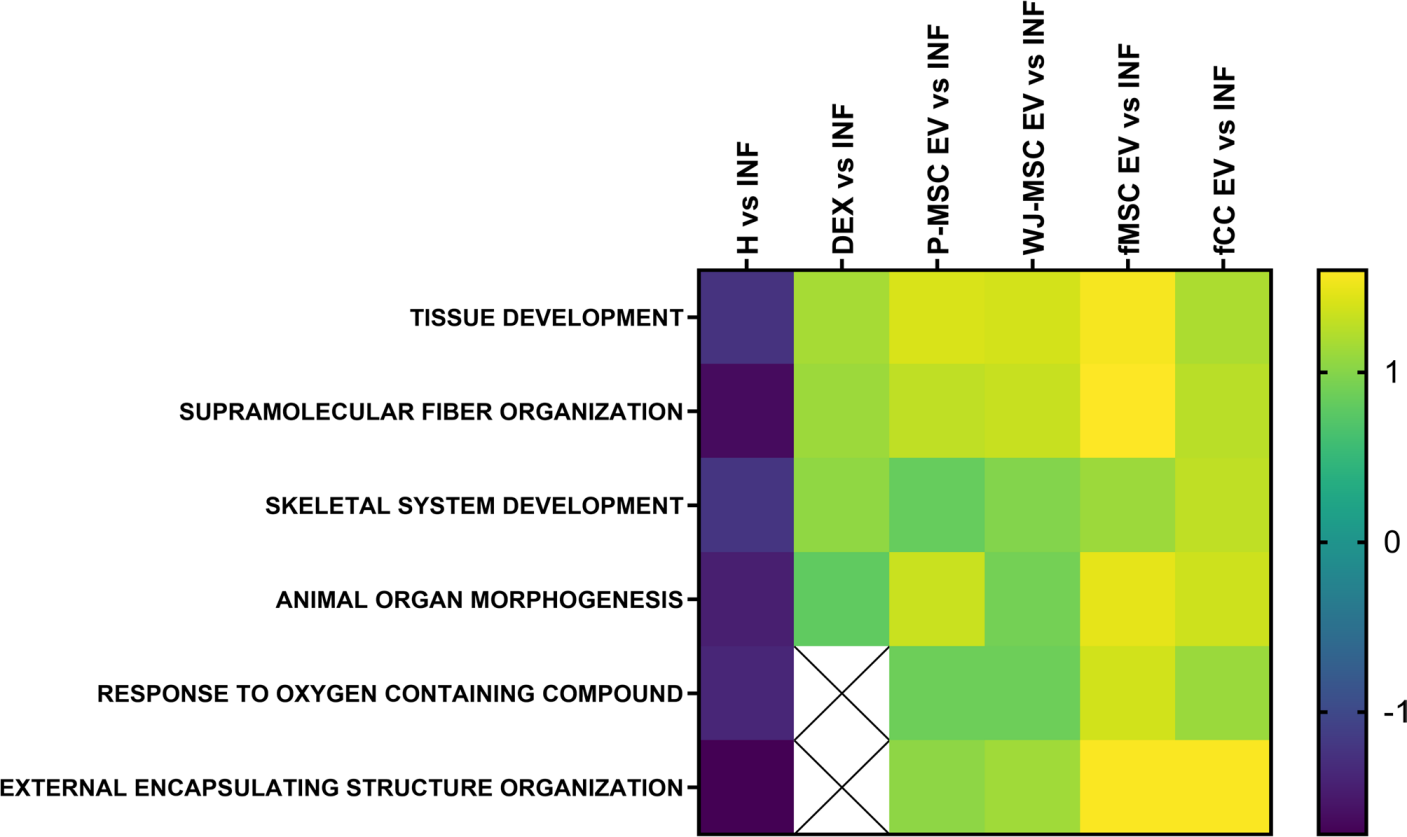
Treatment effect of four different extracellular vesicles on inflamed chondrocytes at 48 h (proteomics data analysis). Protein Set Enrichment analysis of proteins differentially secreted between inflamed untreated and healthy chondrocytes, dexamethasone treated chondrocytes and chondrocytes treated with the four different types of EVs. Analysis of the treatment effect of EVs harvested from ovine fetal articular chondrocytes (fCC EV), ovine fetal umbilical cord blood derived MSCs (fMSC EV), human perinatal placental amnion (P-MSC EV) derived MSCs or human perinatal Wharton’s Jelly (WJ-MSC EV) derived MSCs on inflamed adult articular chondrocytes 48 h after treatment was carried out using Protein Set Enrichment Analysis (PSEA). The heatmap shows a negative enrichment of ECM formation related protein sets like “Tissue development”, “Supramolecular fiber organization”, “Skeletal system development”, “Animal organ morphogenesis” and the defense related protein set “External encapsulating structure organization”. In turn, the same protein sets showed a clear positive enrichment following EV treatment. The results were not significant due to the low annotation level of sheep proteins and hence the low number (85) of genuinely secreted proteins included in the analysis. Ovine adult articular chondrocytes (*n* = 3 biological replicates) with one technical replicate per donor per condition measured twice. Not significant (ns, *p* ≥ 0.05), *p < 0.05, **p < 0.01, ***p < 0.001

Extracellular matrix (ECM)-related proteins such as aggrecan (ACAN), procollagen C-proteinase enhancer (PCOLCE), hyaluronan and proteoglycan link protein 1 (HAPLIN1), collagen type VI alpha 1 (COL6a1), collagen Type V Alpha 2 Chain (COL5A2) and Proteoglycan 4 (PRG4) were non-significantly less abundant in inflamed chondrocytes compared to healthy cells (Figure S9). In turn, treatment with fetal and perinatal EVs increased the abundance of ECM proteins secreted by the inflamed chondrocytes to a variable extent (Figure S9). Protein Set Enrichment Analysis (PSEA) detected a negative enrichment of ECM formation related protein sets like “Tissue development”, “Supramolecular fiber organization”, “Skeletal system development”, “Animal organ morphogenesis” and the defense related protein set “External encapsulating structure organization” (Fig. 7, Table S17). In turn, the same protein sets showed a clear positive enrichment following all treatments (Fig. 7, Table S16). Intriguingly, positive enrichment scores were higher for all four EV treatments compared to dexamethasone treatment. The highest enrichment scores were achieved by fMSC EVs. Unfortunately, due to the low annotation level of sheep proteins only 85 genuinely secreted proteins could be included in the PSEA which hampered statistical evaluation and impeded achieving significant results. Nonetheless, although the results are not significant, they are biologically relevant and mutually reinforcing considering the outcome of the other experiments in this study.

## Discussion

The optimal EV donor cell for OA therapy remains uncertain with both articular chondrocytes and MSCs being plausible sources [27, 32, 34, 46, 47, 67, 89–102]. Since EV cargo largely mirrors the characteristics of the parent cell, EVs derived from cartilage may offer the most relevant cargo for OA treatment [89]. However, as OA is a multifactorial disease affecting all articular tissues, the unique roles of MSCs in immunomodulation and tissue repair suggest that their EVs also carries therapeutically beneficial cargo. Studies reporting superior outcomes with EVs derived from chondrocytes co-cultured with MSCs, compared to chondrocytes alone [89, 103], further suggest that chondrocytes may not necessarily be the most effective EV source. Indeed, in this study, fCC-EVs exhibited the most pronounced therapeutic effects on inflamed ovine chondrocytes, while fetal mesenchymal stem cell-derived EVs (fMSC-EVs) demonstrated the most significant impact on inflamed synoviocytes. These distinct effects suggest that a combinatorial therapeutic approach utilizing a synergistic combination of fCC-EVs and fMSC-EVs may offer a more comprehensive and effective treatment strategy for osteoarthritis (OA). This approach could potentially target both key cellular components of the OA joint – chondrocytes and synoviocytes [104–107] – simultaneously, leading to improved disease modification and a more pronounced overall therapeutic benefit.

EVs exert their functional effects on recipient cells through ligand/receptor interactions at the cell surface or by internalization and transfer of their bioactive cargo [108–115]. Surface molecules not only regulate EVs’ homing, targeting, and uptake by recipient cells but can also directly mediate diverse intercellular signaling functions and elicit functional changes, both with and without membrane fusion [108–115]. However, the specificity of EV- recipient cell interactions remains unresolved. While some studies suggest that EV uptake can occur in any cell type, others indicate that this process is highly selective, requiring specific surface receptors and ligands for effective interaction [111–113, 116–118]. 

In this study, all 4 EV types, the ovine as well as the human derived EVs, effectively interacted with both inflamed and healthy ovine chondrocytes, with no difference between allogeneic and xenogeneic EVs. To clearly discriminate between EV attachment to the cell surface and their actual internalization and account for the limitations of lipophilic dyes that may give non-specific signal in target cells by direct exchange of the dye between both membranes [119–121], EV uptake was verified by detailed confocal imaging. In accordance with previous reports of environmental factors such as cellular stress or the metabolic status affecting EV internalization [111, 116, 117, 122–125], inflammation significantly increased EV uptake in chondrocytes across all fetal and perinatal sources. Notably, uptake of fCC-EVs in articular chondrocytes was significantly higher than that of the three MSCs-derived EVs, supporting emerging evidence of cell-type specific EV uptake with EVs being preferentially taken up by cell types they were originally secreted from [113, 116, 117]. 

Both fetal and perinatal derived EVs, administered at a single dose of 1E + 09 particles/mL, demonstrated anti-inflammatory effects in inflamed chondrocytes and synoviocytes. However, EVs from fetal sources outperformed their perinatal counterparts. Treatment with fetal cell-derived EVs resulted in significantly more differentially expressed genes (DEGs) and enriched pathways than perinatal EVs in both ovine chondrocytes and synoviocytes. Comparative pathway analysis further highlighted the differences in therapeutic efficacy, revealing distinct clustering of fetal versus perinatal EV treatment effects. This age-related clustering was more pronounced than the clustering observed based on EV donor cell type (e.g. chondrocytes versus MSCs), suggesting that the ontogenetic stage of the EV donor cells exerts a stronger influence on the therapeutic impact of EVs than their specific cell type. These findings emphasize the importance of donor age in determining EV functionality and suggest that it may be a critical factor to consider in optimizing EV-based therapies for OA.

In chondrocytes, fCC-EVs induced the highest degree of differential regulation, closely followed by fMSC-EVs. Only DEX and fetal-derived EVs significantly decreased the mRNA levels of MMP12, CHI3L1, CD44, and CFB in inflamed chondrocytes. Comparative pathway analysis underscored the therapeutic distinctions, with fetal-derived EVs showing more pronounced immunomodulatory effects compared to perinatal EVs. Both fMSC-EVs and fCC-EVs downregulated multiple inflammatory pathways, including neutrophil degranulation, neutrophil extracellular trap signaling, and leukocyte extravasation. Notably, all four EV treatments upregulated the anti-senescent Sirtuin signaling pathway in chondrocytes. As ECM-related gene expression typically requires longer periods to manifest [126–128]EV effects on chondrocyte phenotype stability and extracellular matrix production were minimal at T24, limited to a significant downregulation of aggrecan by P-MSC-EVs, while the other EV types non-significantly upregulated this key ECM factor. By T48, fCC-EV and fMSC-EV treatment significantly increased expression of COL2a in inflamed chondrocytes, bringing it closer to healthy control levels, with fCC-EVs also non-significantly increasing aggrecan.

Interestingly, in contrast to the clinically observed prolonged therapeutic effects of corticosteroids, the differential gene regulation in inflamed chondrocytes treated with DEX or any of the EV preparations diminished to single-digit levels by T48. The transient therapeutic effects of EVs on inflamed chondrocytes, lasting approximately 24 h, are consistent with findings from previous studies [125, 129–131], and must be taken in consideration for clinical translation. Further research is required to determine the optimal EV dosage and administration frequency to achieve sustained therapeutic effects.

In synoviocytes, fMSC-EVs achieved the most robust therapeutic effect, with more than twice as many DEGs as the next best treatment, fCC-EVs. While all EV treatments effectively decreased CCL20 levels in inflamed synoviocytes, only DEX and fetal EVs significantly reduced IL6, CD14, CHI3L1 and S100A12. FMSC-EVs and fCC-EVs both downregulated several inflammatory pathways in synoviocytes, including neutrophil degranulation and S100 family signaling. Moreover, fMSC-EVs uniquely upregulated pathways related to extracellular matrix organization and glycosaminoglycan metabolism. In contrast, the two perinatal EVs showed minimal differential gene expression and correspondingly few enriched pathways. Our results suggest that the choice of EV source is a critical factor that can significantly impact the efficacy of EV-based treatments. Further studies are needed to confirm the therapeutic efficacy and translational relevance of the observed extracellular vesicle-mediated effects in an osteoarthritic environment in vivo.

Fetal mammals, unlike adults, are capable of cartilage regeneration during the first two trimesters of gestation [75, 77, 132–136] and demonstrate a rapid resolution of inflammation, which is crucial for successful injury response and regeneration [68, 75, 77, 137]. This inherent regenerative potential suggests that fetal-derived EVs may carry a distinct cargo enriched with factors that promote developmental and regenerative processes, unlike EVs derived from adult tissues, which may have accumulated alterations associated with aging and disease. Furthermore, while EVs derived from adult osteoarthritic (OA) chondrocytes and senescent MSCs can induce a pro-catabolic and pro-inflammatory gene signature in the joints and contribute to pathological processes in articular tissues such as matrix calcification and cellular senescence [55, 138], fetal cells, retaining their developmental phenotype and transcriptomic and proteomic profile [139], have been shown to exert broad-ranging anti-aging effects [49, 140–146], which may be beneficial in age-associated diseases such as OA. Our findings further support these observations, showing significant downregulation of inflammatory and upregulation of ECM markers and pathways in cells treated with fCC-EVs and fMSC-EVs.

Perinatal tissues, such as the placenta and umbilical cord, are typically considered medical waste but are rich sources of MSCs with superior anti-inflammatory and immunosuppressive properties and corresponding therapeutic potential compared to MSCs derived from adult tissues [56, 59–65, 147–153]. Perinatal tissues can be harvested non-invasively and the use of perinatal MSCs and their EVs mitigates many legal, ethical, and moral concerns associated with embryonic or fetal cells, making amnion and Wharton’s jelly attractive sources for regenerative medicine [154–157]. Analogous to the superior immunomodulatory and regenerative capacity of WJ-MSCs compared to P-MSCs for inflamed chondrocytes in this study, WJ-MSCs have shown better proliferation [155] and collagen deposition than P-MSCs in cardiovascular tissue engineering and regenerative medicine applications [154, 155]. 

However, the higher immunomodulatory and regenerative effects observed with EVs from fetal sources in this study support their therapeutic use.

While the use of fetal cell-derived EVs necessitates the consideration of xenogeneic sources, it is important to note that many proteins [158–160] and microRNAs [161–163] are species-specific [164] and even conserved miRNAs, such as the let-7 family, exhibit distinct targeting profiles across species [164–166]. Despite these potential challenges, the successful application of xenogeneic EVs in various preclinical models, including myocardial infarct repair [167, 168] and wound healing [164], demonstrates the feasibility of cross-species EV-mediated therapeutic effects. These studies suggest that EVs derived from the same tissue across different species may carry functionally similar therapeutic cargos [164], supporting the viability of utilizing xenogeneic EVs from optimized cell sources, such as fetal cells, to expand the pool of available donor cell types. Notably, our findings did not reveal significant differences in the uptake of allogeneic and xenogeneic MSC-derived EVs by both ovine and human inflamed chondrocytes, further indicating the potential for successful xenogeneic EV application in OA treatment.

## Conclusions

Selecting the optimal donor cell is essential for tailoring EV cargo and surface molecular profiles to specific therapeutic objectives and maximizing efficacy. In this study, the influence of different EV donor sources on therapeutic efficacy for OA was explored, highlighting significant distinctions between ovine fetal and human perinatal-derived EVs, as well as between fetal chondrocyte (fCC) and fetal MSC (fMSC) sources. Fetal-derived EVs, promoted stronger anti-inflammatory responses and ECM remodeling, such as increased collagen II expression, compared to perinatal EVs in an ovine in vitro model. While fCC-EVs demonstrated the best therapeutic effects on inflamed chondrocytes, fMSC-EVs were most effective on inflamed synoviocytes, suggesting that a combination therapy using EVs from both sources may offer the greatest therapeutic potential.

## Supplementary Information

Below is the link to the electronic supplementary material.

Supplementary Material 1

Supplementary Material 2

Supplementary Material 3

Supplementary Material 4

Supplementary Material 5

Supplementary Material 6

Supplementary Material 7

Supplementary Material 8

Supplementary Material 9

Supplementary Material 10

Supplementary Material 11

Supplementary Material 12

Supplementary Material 13

Supplementary Material 14

Supplementary Material 15

Supplementary Material 16

Supplementary Material 17

Supplementary Material 18

Supplementary Material 19

Supplementary Material 20

Supplementary Material 21

Supplementary Material 22

Supplementary Material 23

Supplementary Material 24

Supplementary Material 25

Supplementary Material 26

Supplementary Material 27

Supplementary Material 28

Supplementary Material 29

Supplementary Material 30

## Data Availability

The datasets generated and analysed during the current study are included in the paper and its supplementary materials.

## Abbreviations

ACAN: Aggrecan
COL: Collagen
CFB: Complement factor B
DAP: Differentially abundant proteins
DEG: Differentially expressed genes
DEX: Dexamethasone
EV: Extracellular vesicle
FC: Fold change
fCC(-EVs): Fetal articular chondrocytes (derived EVs)
FDR: False discovery rate
fMSCs(-EVs): Fetal umbilical cord blood derived MSCs (derived EVs)
FT-FC: Fluorescence-Triggered Flow Cytometry
IL: Interleukin
IPA: Ingenuity pathway analysis
LFQ: Label-free quantification
MMP: Matrix metalloproteinase
MSCs: Mesenchymal stem/stromal cells
MTT: 3-(4,5-dimethylthiazol-2-yl)-2,5-diphenyltetrazolium bromide
NFKBIZ: Nuclear Factor-kappa-B inhibitor zeta
PDE4B: Phosphodiesterase 4B
P-MSCs(-EVs): Perinatal placental amnion derived MCSs (derived EVs)
TFF: Tangential flow filtration
TNF: Tumour necrosis factor
WJ-MSCs(-EVs): Perinatal Wharton’s Jelly derived MSCs (derived EVs)
