# Supplementary material for: Ontogenetic stage and type of donor cells shape extracellular vesicles’ therapeutic potential for osteoarthritis
Source: Stem Cell Res Ther. 2025 Sep 1;16:478. doi: 10.1186/s13287-025-04585-y (PMC12403863; doi:10.1186/s13287-025-04585-y)
Supplement: Supplementary file 2 — Supplementary Material 2 [file 13287_2025_4585_MOESM2_ESM.docx]

**Ontogenetic stage and type of donor cells shape extracellular vesicles’ therapeutic potential for osteoarthritis**

Tarasova K.^1*^, Arteaga M.B.^1*^, Kidtiwong A.^1^, Nivarthi H.^2^, Gamauf J.^2^, Corso G. ^2^, Gültekin S.^1^, Bileck A.^3^, Rothbauer M.^4^, Toegel S.^4^, Hackl M.^5^, Kau-Strebinger S.^6^, Gerner C.^3^, Grillari R.^2,7^, Gerner I.^1,7,+^ and Jenner F.^1,7,+,#^

# Supplementary Methods

Analysis of presence of typical surface marker proteins of WJ-MSC/TERT273 and P-MSC/TERT308

## Prior to seeding into the Hollow Fiber Bioreactor (HFB), the expression of the typical surface marker proteins on WJ-MSC/TERT273 and P-MSC/TERT308 was assessed by performing immunofluorescence staining and a flow cytometric analysis. In detail, we checked for the expression of CD105, CD73 and CD90, and lack expression of CD34, as recommended by position statement on the minimal criteria for defining multipotent mesenchymal stromal cells by the International Society for Cellular Therapy (Dominici et al., 2006; ISCT). Therefore, the following conjugated monoclonal antibodies from BD Pharmingen were used: APC mouse anti-human CD34 (1:10 dilution in blocking solution), APC mouse anti-human CD73 (1:40 dilution in blocking solution), APC mouse anti-human CD90 (1:80 dilution in blocking solution), APC mouse anti-human CD105 (1:40 dilution in blocking solution). 3 × 10^5^ cells were resuspended in PBS, incubated with 200 µL/sample in blocking solution (10% FBS in PBS) for 15 minutes at 37°C, followed by incubation with the monoclonal antibodies for 30 minutes at 37°C protected from light. After a washing step with blocking solution, cells were resuspended in 400 µL of DAPI solution (Roche, Basel, Switzerland) (1:50 dilution in PBS). As a negative control, cells were incubated with the isotype control antibody APC mouse IgG1 (BD Pharmingen, 0.2 mg/mL, 1:40 dilution). Then, the cells were analyzed using the ZE5 Cell analyzer ((BioRad, Hercules, USA) by setting a gate at 10,000 events for viable cells. Data analysis was performed using Kaluza Analysis 2.1 software.

## Characterization of the fetal MSC isolated from the Hollow Fiber Bioreactor

After six weeks of culture, fMSCs were aseptically isolated by breaking the cartridge of the HFB. The fibers the cells were growing on were transferred into 6-well plates and cultured in complete StemMACS medium supplemented with media supplement XF and 1% Pen/Strep under standard conditions. Differentiation experiments were conducted in technical duplicates and maintained for three weeks. fMSCs isolated from monolayer culture under standard conditions served as controls.

### Trilineage Differentiation

For chondrogenic differentiation, 300.000 cells were suspended in 1 mL of chondrogenic differentiation medium (StemPro Chondrogenesis Differentiation Kit; Gibco) and centrifuged for 5 min at 280 g, 4^o^C to form a pellet. The cell pellets were cultured in duplicate in 15mL falcon tubes and were carefully shaken daily to prevent attachment to the plastic tube. Medium was changed twice a week. After 3 weeks of cultivation in differentiation medium, the pellets were fixed with buffered 4% formalin (ACM, Herba Chemosan Apotheker AG) and subjected to paraffin embedding and sectioning. The sections were stained with Alcian blue (Sanova, Vienna, Austria) according to a standard staining protocol to confirm the production of acidic mucosubstances [1].

Osteogenic differentiation was performed in monolayer using the osteogenic differentiation medium (StemPro Osteogenesis Differentiation Kit; Gibco). First, 3000 cells/well were seeded on 24-well plates in standard culture medium. After 24h, the medium was replaced by osteogenic differentiation medium and changed twice a week. After 3 weeks, the cells were fixed with buffered 4% formalin and stained for extracellular Calcium deposition using von Kossa staining (silver nitrate, Merck; Natriumthiosulfat, Sigma Aldrich) according to a standard staining protocol [1].

Adipogenic differentiation was performed in monolayer culture by seeding 4000 cells/well in 24-well plates with standard culture medium. After 24 hours, adipogenic differentiation was induced using StemMACS AdipoDiff Medium (Miltenyi Biotec). The medium was changed twice weekly. After 3 weeks, cells were fixed with 4% buffered formalin and stained with Oil Red O (Sigma Aldrich) to visualize intracellular lipid vacuoles following a standard staining protocol [1].

### Flow Cytometry

For the flow cytometry, cells were trypsinised at passage 3, and 1 × 10^5^ cells per sample were washed with PBS +/+ supplemented with 2% FCS (FACS Buffer) (Capricon Scientific, FBS-12B, Germany). Single stains were prepared by incubating the following primary monoclonal antibodies and respective isotype controls for 15 minutes on ice. Cells were washed twice with FACS buffer. Cells stained with purified antibodies were incubated with secondary antibody for 10 minutes and washed twice with FACS buffer. A total of 1 × 10^4^ events were acquired via FACS Canto II and analyzed with Flow Jo 10.8.0 Software (BD Biosciences, New Jersey, USA). APC-CD29 (Clone MAR4, mouse anti human IgG1,κ, 1:10, Biorad, Hercules, CA, USA), FITC-CD44 (Clone 25.32, mouse anti sheep IgG1,κ, 1:200, Biorad, Hercules, CA, USA), PE-CD166 (Clone 3A6, mouse anti human IgG1,κ 1:10, BD Biosciences, New Jersey, USA), Purified CD45 (Clone 1.11.32, IgG1, 1:50, Biorad, Hercules, CA, USA). Purified-CD31 (Clone CO.3E1D4, mouse anti sheep IgG2a, 1:50, Biorad, Hercules, CA, USA), Secondary Antibody FITC (Polyclonal IgG, goat anti mouse, IgG, 1/200, Biorad, Hercules, CA, USA).

## Characterization of the fetal Chondrocytes isolated from the Hollow Fiber Bioreactor

After six weeks of culture, fCCs were aseptically isolated by breaking the cartridge of the HFB similarly to fMSCs. The fibers the cells were growing on were transferred into 6-well plates and cultured in complete StemMACS medium supplemented with media supplement XF and 1% Pen/Strep under standard conditions. Chondrogenic differentiation experiments were conducted in technical duplicates and maintained for 21 days. FCCs isolated from monolayer culture under standard conditions served as controls.

For chondrogenic differentiation, 300.000 cells were suspended in 1 mL of in complete StemMACS medium supplemented with media supplement XF and 1% Pen/Strep and centrifuged for 5 min at 280 g, 4^o^C to form a pellet. The cell pellets were cultured in duplicate in 15mL falcon tubes and were carefully shaken daily to prevent attachment to the plastic tube. Medium was changed twice a week. After 21 days of cultivation, the pellets were fixed with buffered 4% formalin (ACM, Herba Chemosan Apotheker AG) and subjected to paraffin embedding and sectioning. The sections were stained with Alcian blue (Sanova, Vienna, Austria) according to a standard staining protocol to confirm the production of acidic mucosubstances [1].

# Supplementary Results

Analysis of presence of typical surface marker proteins of WJ-MSC/TERT273 and P-MSC/TERT308

Both WJ-MSC/TERT273 and P-MSC/TERT308 were positive for MSCs markers CD73, CD90 and CD105, and negative for the hematopoietic progenitor cell antigen CD34 (Figure S1).

## Characterization of the fetal MSC isolated from the Hollow Fiber Bioreactor

Both bioreactor-derived and monolayer-cultured fMSCs were positive for MSC markers CD44 and CD166, and negative for CD31 and CD45 (Figure S2A), isotype controls were negative. Additionally, bioreactor-isolated fMSCs exhibited osteogenic and chondrogenic differentiation potential after three weeks in culture evidenced by the Von Kossa staining of Calcium deposits and Alcian blue stainning of preoteoglycans, respectively (Figure S2B). Analogous to previous studies using fetal cells [2–7], the fMSCs did not show adipogenic differentiation, which may be due to the reduced activation of the proliferator–activated receptor g (PPAR-c) pathway in fetal cells [2–7].

## Characterization of the fetal Chondrocytes isolated from the Hollow Fiber Bioreactor

# Both bioreactor-derived and monolayer-cultured fCCs exhibited chondrogenic potential after 21 days in culture evidenced by the Alcian blue stainning of proteoglycans (Figure S2C).

# References

1 Mulisch M, Welsch U, editors. Romeis - Mikroskopische Technik. 19th ed. Springer Berlin Heidelberg, 2015.

2 Ragni E, Viganò M, Parazzi V, et al. Adipogenic potential in human mesenchymal stem cells strictly depends on adult or foetal tissue harvest. The International Journal of Biochemistry & Cell Biology 2013;45:2456–2466.

3 Ribitsch I, Chang-Rodriguez S, Egerbacher M, et al. Sheep Placenta Cotyledons: A Noninvasive Source of Ovine Mesenchymal Stem Cells. Tissue Engineering Part C, Methods 2017;23:298–310.

4 Reed SA, Johnson SE. Equine umbilical cord blood contains a population of stem cells that express Oct4 and differentiate into mesodermal and endodermal cell types. Journal of Cellular Physiology 2008;215:329–336.

5 Kern S, Eichler H, Stoeve J, et al. Comparative Analysis of Mesenchymal Stem Cells from Bone Marrow, Umbilical Cord Blood, or Adipose Tissue. Stem Cells 2006;24:1294–1301.

6 Shetty P, Cooper K, Viswanathan C. Comparison of proliferative and multilineage differentiation potentials of cord matrix, cord blood, and bone marrow mesenchymal stem cells. Asian J Transfus Sci 2010;4:14–24.

7 Wagner W, Wein F, Seckinger A, et al. Comparative characteristics of mesenchymal stem cells from human bone marrow, adipose tissue, and umbilical cord blood. Exp Hematol 2005;33:1402–1416.
