# Supplementary material for: Ontogenetic stage and type of donor cells shape extracellular vesicles’ therapeutic potential for osteoarthritis
Source: Stem Cell Res Ther. 2025 Sep 1;16:478. doi: 10.1186/s13287-025-04585-y (PMC12403863; doi:10.1186/s13287-025-04585-y)
Supplement: Supplementary file 3 — Supplementary Material 3 [file 13287_2025_4585_MOESM3_ESM.docx]

**Ontogenetic stage and type of donor cells shape extracellular vesicles’ therapeutic potential for osteoarthritis**

**Supplementary Figure Legends**

Figure S1: Characterisation of WJ-MSC/TERT273 and P-MSC/TERT308 showing canonical surface marker expression of MSCs.

Both WJ-MSC/TERT273 and P-MSC/TERT308 were positive for MSCs markers CD73, CD90 and CD105, and negative for the hematopoietic progenitor cell antigen CD34, isotype controls were negative (one representative FT-FC experiment is shown).

Figure S2: Characterisation of the ovine fetal articular chondrocytes (fCC), ovine fetal umbilical cord blood derived mesenchymal stromal cells (fMSC) showing trilineage differentiation capacity and canonical surface marker expression of ovine MSCs.

(A) Both bioreactor-derived and monolayer-cultured fMSC were positive for MSCs markers CD44 and CD166, and negative for CD31 and CD45, isotype controls were negative (one representative FT-FC experiment is shown). However, due to the use of ovine donors in this study, the availability of validated antibodies against specific MSCs markers- CD73, CD90 and CD105- for sheep is currently limited, which precluded their inclusion in the flow cytometry panel.

(B) Both bioreactor-derived and monolayer-cultured fMSC exhibited chondrogenic (stained with Alcian blue) and osteogenic (stained with von Kossa stain) differentiation potential after three weeks in culture, but not adipogenic differentiation (one representative experiment is shown).

(C) Both bioreactor-derived and monolayer-cultured fCC presence of proteoglycans (stained with Alcian blue) (one representative experiment is shown).

Figure S3: Western blot analysis of whole-cell lysates and TFF-enriched fetal EVs showed the presence of tetraspanins CD81 (A) and CD9 (B) in both fetal cell-derived EVs, while calnexin (C), a non-exosomal marker, was only detected in the cell lysate.

(A) Full-length blots analysis of CD81 (25 kDa) marker in 1: molecular weight marker; 2: TFF enriched EV derived from fMSC cultured in hollow fiber bioreactor; 3: TFF enriched EV derived from fCC cultured in hollow fiber bioreactor; 4: TFF enriched EV derived from fMSC cultured in monolayer; 5: TFF enriched EV derived from fCC cultured in monolayer; 6: fCC whole-cell lysates

(B) Full-length blots analysis of CD9 (25 kDa) marker in 1: molecular weight marker; 2: TFF enriched EV derived from fMSC cultured in hollow fiber bioreactor; 3: TFF enriched EV derived from fCC cultured in hollow fiber bioreactor; 4: TFF enriched EV derived from fMSC cultured in monolayer; 5: TFF enriched EV derived from fCC cultured in monolayer; 6: fCC whole-cell lysates.

(C) Full-length blots analysis of Calnexin (90 kDa) marker in 1: molecular weight marker; 2: TFF enriched EV derived from fMSC cultured in hollow fiber bioreactor; 3: TFF enriched EV derived from fCC cultured in hollow fiber bioreactor; 4: TFF enriched EV derived from fMSC cultured in monolayer; 5: TFF enriched EV derived from fCC cultured in monolayer; 6: fCC whole-cell lysates.

Figure S4: Uptake of ovine fetal articular chondrocytes (fCC-EVs), ovine fetal umbilical cord blood derived MSCs (fMSC-EV), human perinatal placental amnion derived MSCs (P-MSC-EV) or human perinatal Wharton’s Jelly derived MSCs (WJ-MSC-EV) derived extracellular vesicles (EV) in human inflamed and ovine inflamed and healthy adult articular chondrocytes.

(A) Scatter plots (mean ± SD) showing Red Integrated Intensity (RCU µm^2^/Image) of inflamed adult human articular chondrocytes. All four EV treatments were successfully taken up by human inflamed articular chondrocytes at 24h and a significant increase was observed on fetal EV treatments over perinatal (fMSC EV vs P-MSC EV p=0.0078, fMSC EV vs WJ-MSC EV p=0.0098; fCC EV vs P-MSC EV p=0.0005, fCC EV vs WJ-MSC EV p=0.0007).

(B) Scatter plots (mean ± SD) showing Red Integrated Intensity (RCU µm2/Image) of inflamed adult human articular chondrocytes. All four EV treatments were successfully taken up by human inflamed chondrocytes within the period of 24h. Fetal EVs were taken up significantly (p < 0.0001) higher than perinatal EVs (fMSC or fCC EV vs P-MSC or WJ-MSC EV).

(C) Scatter plots (mean ± SD) showing Red Integrated Intensity (RCU µm2/Image) of inflamed cells over the period of 24h. EVs labelled only, dye only and EVs non labelled were used as controls. A consistent unchanged RCU signal was observed in all conditions in the absence of cells within a 24h period.

(D- E) Scatter plots (mean ± SD) showing percentage of positive cells at 1h, 6h and 24h incubation period. Flow Cytometry analysis revealed increased fluorescent cell counts following 1 hour of EV incubation, which continued over 24 hours. fCC EV were significantly higher taken up in both (D) healthy (p=0.0255) and (E) inflamed cells (p=0.0469) compared to fMSC EV. Ovine adult articular chondrocytes (n = 3 biological replicates) with two technical replicates per donor per condition.

Not significant (ns, p≥0.05), *p<0.05, **p <0.01, ***p<0.001.

Figure S5: Treatment effect of ovine fetal articular chondrocytes (fCC-EV), ovine fetal umbilical cord blood derived MSCs (fMSC-EV), human perinatal placental amnion derived MSCs (P-MSC-EV) or human perinatal Wharton’s Jelly derived MSCs (WJ-MSC-EV) derived extracellular vesicles (EV) on inflamed ovine adult articular chondrocytes and synoviocytes (Metabolic activity analysis (MTT assay), Proliferation and Wound healing assays).

(A-D) Scatter plots (mean ± SD) showing metabolic activity of healthy and inflamed chondrocytes (A and B) and synoviocytes (C and D), illustrated as MTT values (percentage of healthy (%)), was not significantly affected by (A) either fetal or perinatal derived EVs or DEX (40nM) treatments at 24h, (B) but significantly increased with P-MSC EV (p = 0.0014) compared to inflamed untreated articular chondrocytes at 48h. (C) Neither fetal or perinatal derived EVs or DEX (40nM) treatments had no significant influence on metabolic activity of inflamed synoviocytes at 24h, (D) but both fetal-derived EVs significantly increased the viability of inflamed synoviocytes at 48 hours (fMSC EV: p = 0.0065; fCC EV: p = 0.0232) compared to inflamed untreated ovine adult synoviocytes.

(E-H) Neither fetal nor perinatal-derived EVs significantly affected cell proliferation (percentage of phase area confluence (%)) or wound healing (percentage of wound width (%)) in inflamed articular chondrocytes (E and F) or synoviocytes (G and H). Ovine adult articular chondrocytes and synoviocytes (n = 3 biological replicates) with three (for MTT and wound healing assays) to six (proliferation assays) technical replicates per donor per condition. Not significant (ns, p≥0.05), *p<0.05, **p <0.01, ***p<0.001.

Figure S6: Venn Diagrams of differentially expressed genes (DEGs) at 24h (T24) between ovine fetal articular chondrocytes (fCC-EV), ovine fetal umbilical cord blood derived MSCs (fMSC-EV), human perinatal placental amnion derived MSCs (P-MSC-EV) or human perinatal Wharton’s Jelly derived MSCs (WJ-MSC-EV) derived extracellular vesicles (EV) and inflamed untreated adult articular chondrocytes.

(A-C) Venn diagram of the genes differentially regulated (adjusted p < 0.05) between healthy and inflamed untreated (H vs INF), inflamed P-MSC-EV and inflamed untreated (P-MSC-EV vs INF), inflamed WJ-MSC-EV and inflamed untreated (WJ-MSC-EV vs INF), inflamed fCC-EV and inflamed untreated (fCC-EV vs INF), inflamed fMSC-EV and inflamed untreated (fMSC-EV vs INF) and Inflamed DEX and Inflamed untreated (DEX vs INF) at T24 (A) overall and divided into (B) up- and (C) down-regulated DEGs. Inflammatory stimulation of chondrocytes resulted in 56 DEGs (43 upregulated, 13 downregulated) at T24 compared to healthy cells. Treatment of inflamed chondrocytes at T24 with WJ-MSC-EVs led to 69 DEGs (9 upregulated, 60 downregulated), P-MSC-EVs resulted in 57 DEGs (23 upregulated, 34 downregulated), fMSC-EVs led to 85 DEGs (9 upregulated, 76 downregulated), fCC-EVs resulted in 89 DEGs (9 upregulated, 80 downregulated), and DEX treatment resulted in 168 DEGs (69 upregulated, 99 downregulated) compared to untreated inflamed cells.

Figure S7: Venn Diagrams of differentially expressed genes (DEGs) at 48h (T48) between ovine fetal articular chondrocytes (fCC-EV), ovine fetal umbilical cord blood derived MSCs (fMSC-EV), human perinatal placental amnion derived MSCs (P-MSC-EV) or human perinatal Wharton’s Jelly derived MSCs (WJ-MSC-EV) derived extracellular vesicles (EV) and inflamed untreated ovine adult articular chondrocytes.

(A-C) Venn diagram of the genes differentially regulated (adjusted p < 0.05) between healthy and inflamed untreated (H vs INF), inflamed P-MSC-EVs and inflamed untreated (P-MSC-E vs INF), inflamed WJ-MSC-EV and inflamed untreated (WJ-MSC-EVs vs INF), inflamed fCC-EV and inflamed untreated (fCC-EVs vs INF), inflamed fMSC-EV and inflamed untreated (fMSC-EV vs INF) and Inflamed DEX and Inflamed untreated (DEX vs INF) at T48 (A) overall and divided into (B) up- and (C) down-regulated DEGs. Inflammatory stimulation of chondrocytes resulted in 111 DEGs (56 upregulated, 55 downregulated) at T48 compared to healthy cells. Treatment of inflamed chondrocytes at T48 with WJ-MSC-EV led to 1 downregulated DEG, P-MSC-EV resulted in 7 DEGs (4 upregulated, 3 downregulated), fMSC-EV led to 2 DEGs (1 upregulated, 1 downregulated), fCC-EV resulted in 1 upregulated DEG, and DEX treatment resulted in 5 DEGs (4 upregulated, 1 downregulated) compared to untreated inflamed cells.

Figure S8: Venn Diagrams of differentially expressed genes (DEGs) at 48h (T48) between ovine fetal articular chondrocytes (fCC-EV), ovine fetal umbilical cord blood derived MSCs (fMSC-EV), human perinatal placental amnion derived MSCs (P-MSC-EV) or human perinatal Wharton’s Jelly derived MSCs (WJ-MSC-EV) derived extracellular vesicles (EV) and inflamed untreated ovine adult synoviocytes.

Venn diagram of the genes differentially regulated (adjusted p < 0.05) between healthy and inflamed untreated (H vs INF), inflamed P-MSC-EVs and inflamed untreated (P-MSC-EV vs INF), inflamed WJ-MSC-EV and inflamed untreated (WJ-MSC-EVs vs INF), inflamed fCC-EV and inflamed untreated (fCC-EV vs INF), inflamed fMSC-EV and inflamed untreated (fMSC-EV vs INF) and Inflamed DEX and Inflamed untreated (DEX vs INF) at T48 (A) overall and divided into (B) up- and (C) down-regulated DEGs. Inflammatory stimulation of synoviocytes resulted in 134 DEGs (87 up- and 47 downregulated) compared to the healthy control at T48. Treatment of inflamed synoviocytes at T48 with WJ-MSC derived EV led to 7 DEGs (4 up- and 3 downregulated), P-MSC derived EV resulted in 16 DEGs (11 up- and 5 downregulated), fMSC derived EV led to 98 DEGs (28 up- and 70 downregulated), fCC derived EV resulted in 43 DEGs (18 up- and 25 downregulated) and with DEX resulted in 109 DEGs (67 up- and 42 downregulated) compared to inflamed untreated cells.

## Figure S9: Treatment effect of four different extracellular vesicles (EVs) on inflamed chondrocytes at 24h and 48h (proteomic analysis).

(A) Scatter plots (mean ± SD) showing Extracellular matrix (ECM)-related proteins such as aggrecan (ACAN), procollagen C-proteinase enhancer (PCOLCE), hyaluronan and proteoglycan link protein 1 (HAPLIN1), collagen type VI alpha 1 (COL6a1), collagen Type V Alpha 2 Chain (COL5A2) and Proteoglycan 4 (PRG4) were non significantly less abundant in inflamed chondrocytes compared to healthy cells. Treatment with fetal and perinatal EVs increased the abundance of ECM proteins secreted by the inflamed chondrocytes to a variable extent. fCC-EV treatment significantly (q=0.000) increased Col6A1secretion compared to healthy cells and WJ-MSC-EVs significantly increased the abundance of PRG4 compared to healthy cells. P-MSC EV achieved the least increase in the secretion of ECM proteins such as ACAN (q=0.05), PCOLCE (q=0.000), COL6A1 (q=0.000), and COL5A2 (q= 0.02) compared to the other EV treatments. Ovine adult articular chondrocytes (n = 3 biological replicates) with one technical replicate per donor per condition. Not significant (ns, p≥0.05), *p<0.05, **p <0.01, ***p<0.001.

**Supplementary Table Legends**

Table S1: Genes commonly or uniquely differentially expressed in ovine adult articular chondrocytes in response to the different treatments (healthy (H), inflamed untreated (INF), inflamed treated with dexamethasone (DEX) or with EVs harvested from ovine fetal articular chondrocytes (fCC-EVs), ovine fetal umbilical cord blood derived MSCs (fMSC-EVs), human perinatal placental amnion derived MSCs (P-MSC-EVs) or human perinatal Wharton’s Jelly-derived MSCs (WJ-MSC-EVs) 24h after treatment.

Table S2: Genes commonly or uniquely differentially expressed in ovine adult articular chondrocytes between different EV treatments with EVs harvested from ovine fetal articular chondrocytes (fCC-EVs), ovine fetal umbilical cord blood derived MSCs (fMSC-EVs), human perinatal placental amnion derived MSCs (P-MSC-EVs) or human perinatal Wharton’s Jelly-derived MSCs (WJ-MSC-EVs) 24h after treatment.

Table S3: Genes commonly or uniquely differentially expressed in ovine adult articular chondrocytes at 48h in response to the different treatments (healthy (H), inflamed untreated (INF), inflamed treated with dexamethasone (DEX) or with EVs harvested from ovine fetal articular chondrocytes (fCC-EVs), ovine fetal umbilical cord blood derived MSCs (fMSC-EVs), human perinatal placental amnion derived MSCs (P-MSC-EVs) or human perinatal Wharton’s Jelly-derived MSCs (WJ-MSC-EVs).

Table S4: Genes commonly or uniquely differentially expressed in ovine adult articular chondrocytes between different EV treatments with EVs harvested from ovine fetal articular chondrocytes (fCC-EVs), ovine fetal umbilical cord blood derived MSCs (fMSC-EVs), human perinatal placental amnion derived MSCs (P-MSC-EVs) or human perinatal Wharton’s Jelly-derived MSCs (WJ-MSC-EVs) 48h after treatment.

Table S5: Genes commonly or uniquely differentially expressed in ovine adult synviocytes in response to the different treatments (healthy (H), inflamed untreated (INF), inflamed treated with dexamethasone (DEX) or with EVs harvested from ovine fetal articular chondrocytes (fCC-EVs), ovine fetal umbilical cord blood derived MSCs (fMSC-EVs), human perinatal placental amnion derived MSCs (P-MSC-EVs) or human perinatal Wharton’s Jelly-derived MSCs (WJ-MSC-EVs) 48h after treatment.

Table S6: Genes commonly or uniquely differentially expressed in ovine adult synoviocytes between different EV treatments with EVs harvested from ovine fetal articular chondrocytes (fCC-EVs), ovine fetal umbilical cord blood derived MSCs (fMSC-EVs), human perinatal placental amnion derived MSCs (P-MSC-EVs) or human perinatal Wharton’s Jelly-derived MSCs (WJ-MSC-EVs) 48h after treatment.

Table S7: Canonical pathways enriched in genes differentially expressed in ovine adult articular chondrocytes at 24h after  treatment with EVs harvested from ovine fetal articular chondrocytes (fCC-EV), ovine fetal umbilical cord blood derived MSCs (fMSC-EVs), human perinatal Wharton’s Jelly (WJ-MSC-Evs) or amnion (P-MSC-EVs) derived MSCs compared to untreated inflamed chondrocytes (sorted by ascending absolute z-score).

Table S8: Canonical Pathways enriched in genes differentially expressed in ovine adult synoviocytes at 48h after treatment with EVs harvested from ovine fetal articular chondrocytes (fCC-EVs), ovine fetal umbilical cord blood derived MSCs (fMSC-EVs), human perinatal Wharton’s Jelly (WJ-MSC-EVs) or amnion (P-MSC-EVs) derived MSCs compared to untreated inflamed chondrocytes (sorted by ascending absolute z-score).

Table S9: Differentially expressed genes contributing to the activation or inhibition of canonical pathways identified by Ingenuity Pathway Analysis in ovine adult articular chondrocytes at 24h after treatment with EVs harvested from human perinatal amnion (P-MSC-EVs) derived MSCs compared to untreated inflamed adult articular chondrocytes.

Table S10: Differentially expressed genes contributing to the activation or inhibition of canonical pathways identified by Ingenuity Pathway Analysis in ovine adult articular chondrocytes at 24h after treatment with EVs harvested from human perinatal Wharton’s Jelly (WJ-MSC-EVs) derived MSCs compared to untreated inflamed adult articular chondrocytes.

Table S11: Differentially expressed genes contributing to the activation or inhibition of canonical pathways identified by Ingenuity Pathway Analysis in ovine adult articular chondrocytes at 24h after treatment with EVs harvested from ovine fetal umbilical cord blood derived MSCs (fMSC-EVs) compared to untreated inflamed adult articular chondrocytes.

Table S12: Differentially expressed genes contributing to the activation or inhibition of canonical pathways identified by Ingenuity Pathway Analysis in ovine adult articular chondrocytes at 24h after treatment with EVs harvested from ovine fetal articular chondrocytes (fCC-EVs) compared to untreated inflamed adult articular chondrocytes.

Table S13: Differentially expressed genes contributing to the activation or inhibition of canonical pathways identified by Ingenuity Pathway Analysis in ovine adult articular chondrocytes at 48h after treatment with EVs harvested from human perinatal amnion (P-MSC-EVs) derived MSCs compared to untreated inflamed adult synoviocytes.

Table S14: Differentially expressed genes contributing to the activation or inhibition of canonical pathways identified by Ingenuity Pathway Analysis in ovine adult articular chondrocytes at 48h after treatment with EVs harvested from human perinatal Wharton’s Jelly (WJ-MSC-EVs) derived MSCs compared to untreated inflamed adult synoviocytes.

Table S15: Differentially expressed genes contributing to the activation or inhibition of canonical pathways identified by Ingenuity Pathway Analysis in ovine adult articular chondrocytes at 48h after treatment with EVs harvested from ovine fetal umbilical cord blood derived MSCs (fMSC-EVs) compared to untreated inflamed adult synoviocytes.

Table S16: Differentially expressed genes contributing to the activation or inhibition of canonical pathways identified by Ingenuity Pathway Analysis in ovine adult articular chondrocytes at 48h after treatment with EVs harvested from ovine fetal articular chondrocytes (fCC-EVs) compared to untreated inflamed adult synoviocytes.

Table S17: Protein Set Enrichment Analysis (PSEA) carried out for the genuinely secreted proteins identified from EV treated (ovine fetal articular chondrocytes (fCC-EVs), ovine fetal umbilical cord blood derived MSCs (fMSC-EVs), human perinatal placental amnion derived MSCs (P-MSC-EVs) or human perinatal Wharton’s Jelly-derived MSCs (WJ-MSC-EVs)), dexamethasone treated, and healthy compared to the inflamed untreated cells 48h after treatment.
