# Supplementary figures and images for: Ontogenetic stage and type of donor cells shape extracellular vesicles’ therapeutic potential for osteoarthritis
Source: Stem Cell Res Ther. 2025 Sep 1;16:478. doi: 10.1186/s13287-025-04585-y (PMC12403863; doi:10.1186/s13287-025-04585-y)

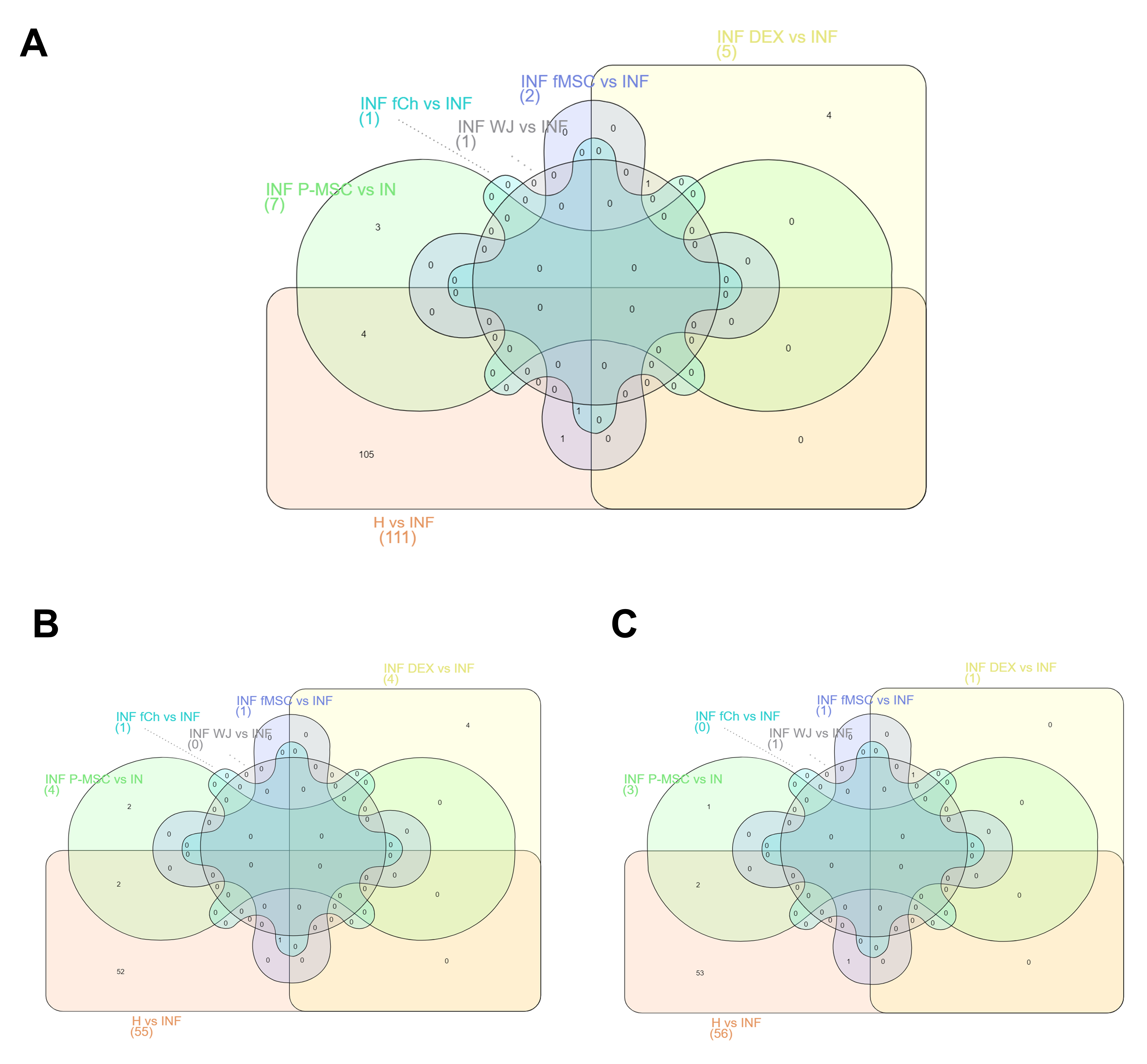

Supplement: Supplementary file 19 — Supplementary Material 19 [file 13287_2025_4585_MOESM19_ESM.tiff]

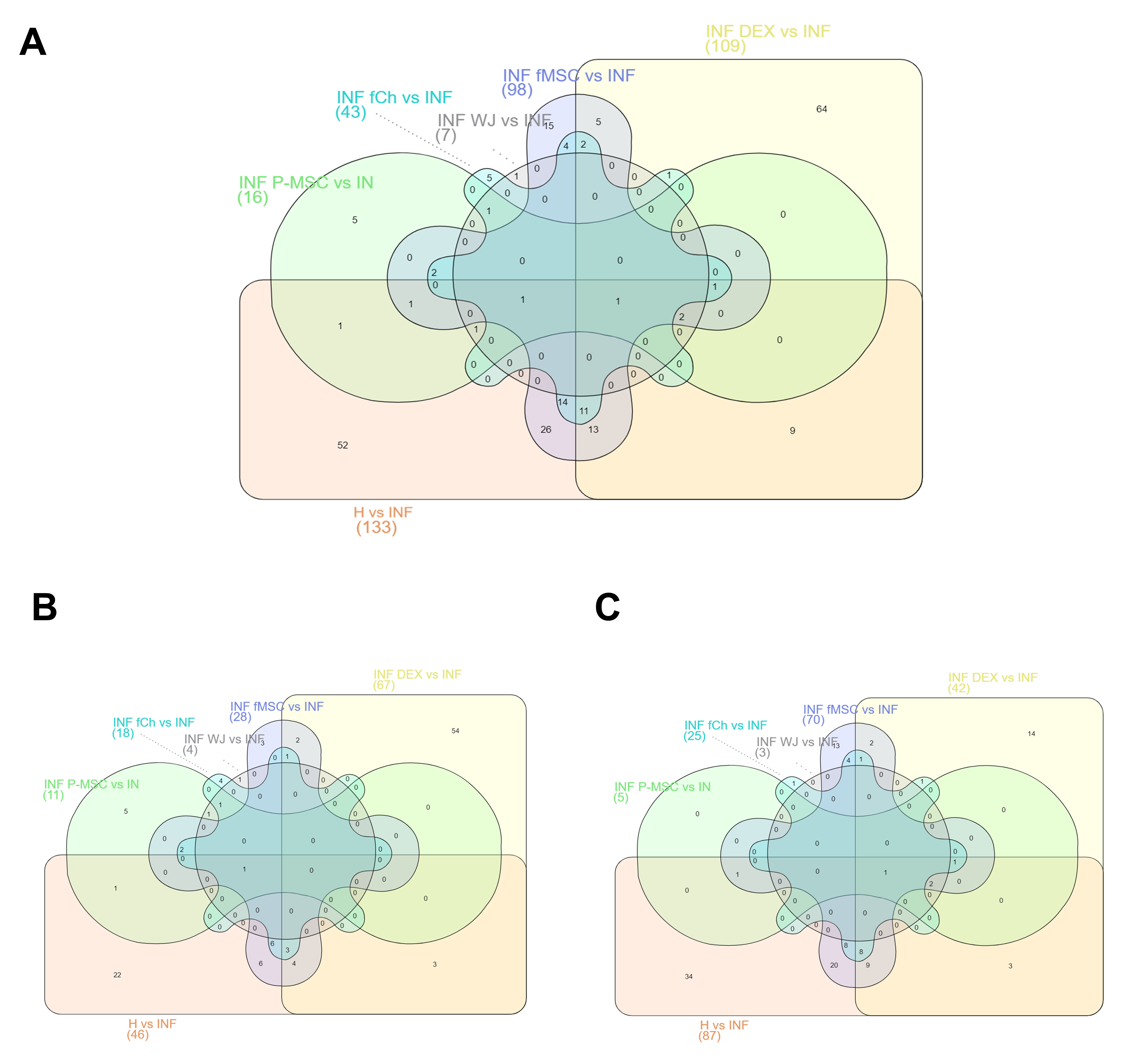

Supplement: Supplementary file 20 — Supplementary Material 20 [file 13287_2025_4585_MOESM20_ESM.tiff]

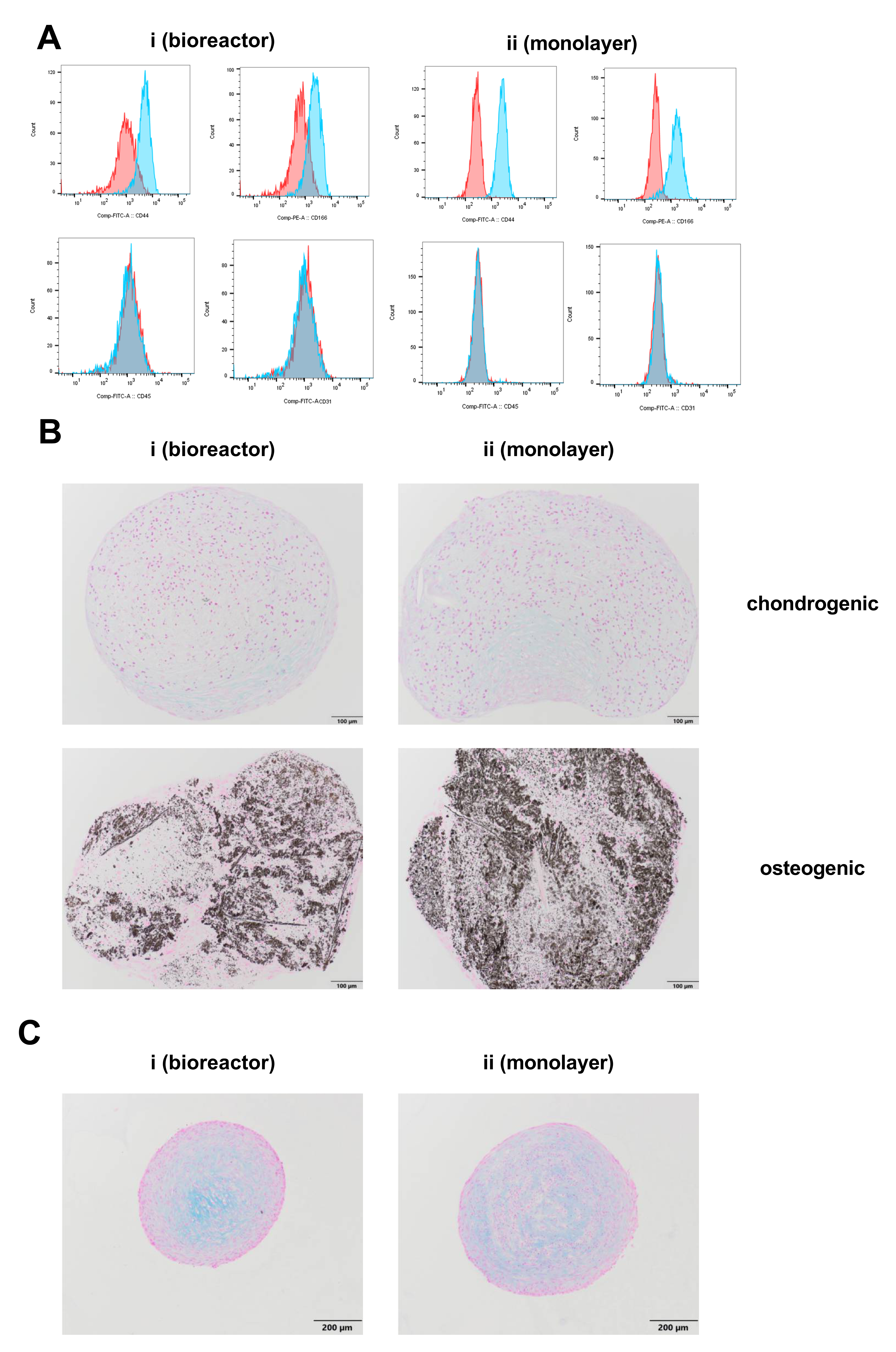

Supplement: Supplementary file 26 — Supplementary Material 26 [file 13287_2025_4585_MOESM26_ESM.tiff]

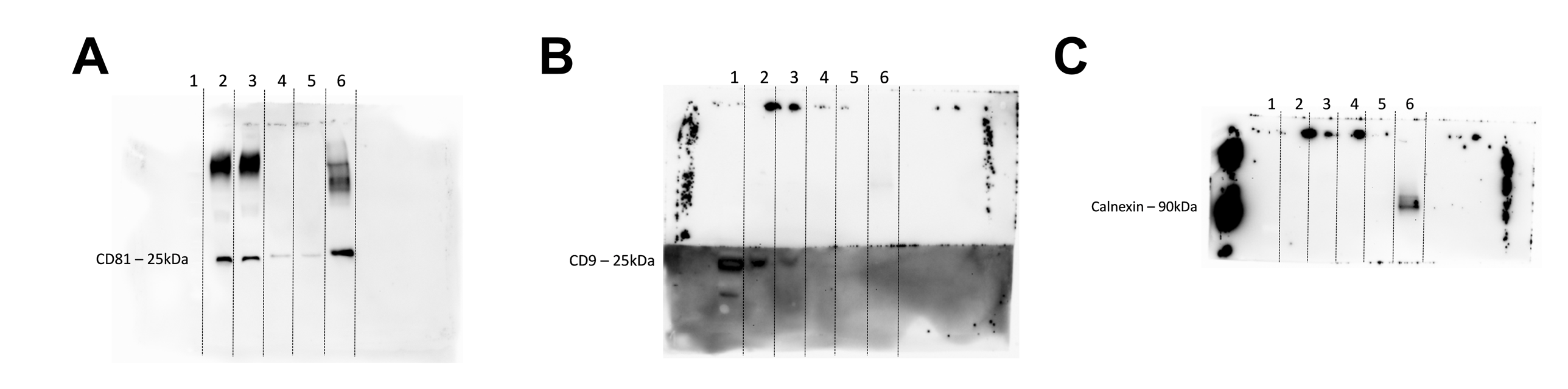

Supplement: Supplementary file 27 — Supplementary Material 27 [file 13287_2025_4585_MOESM27_ESM.tif]

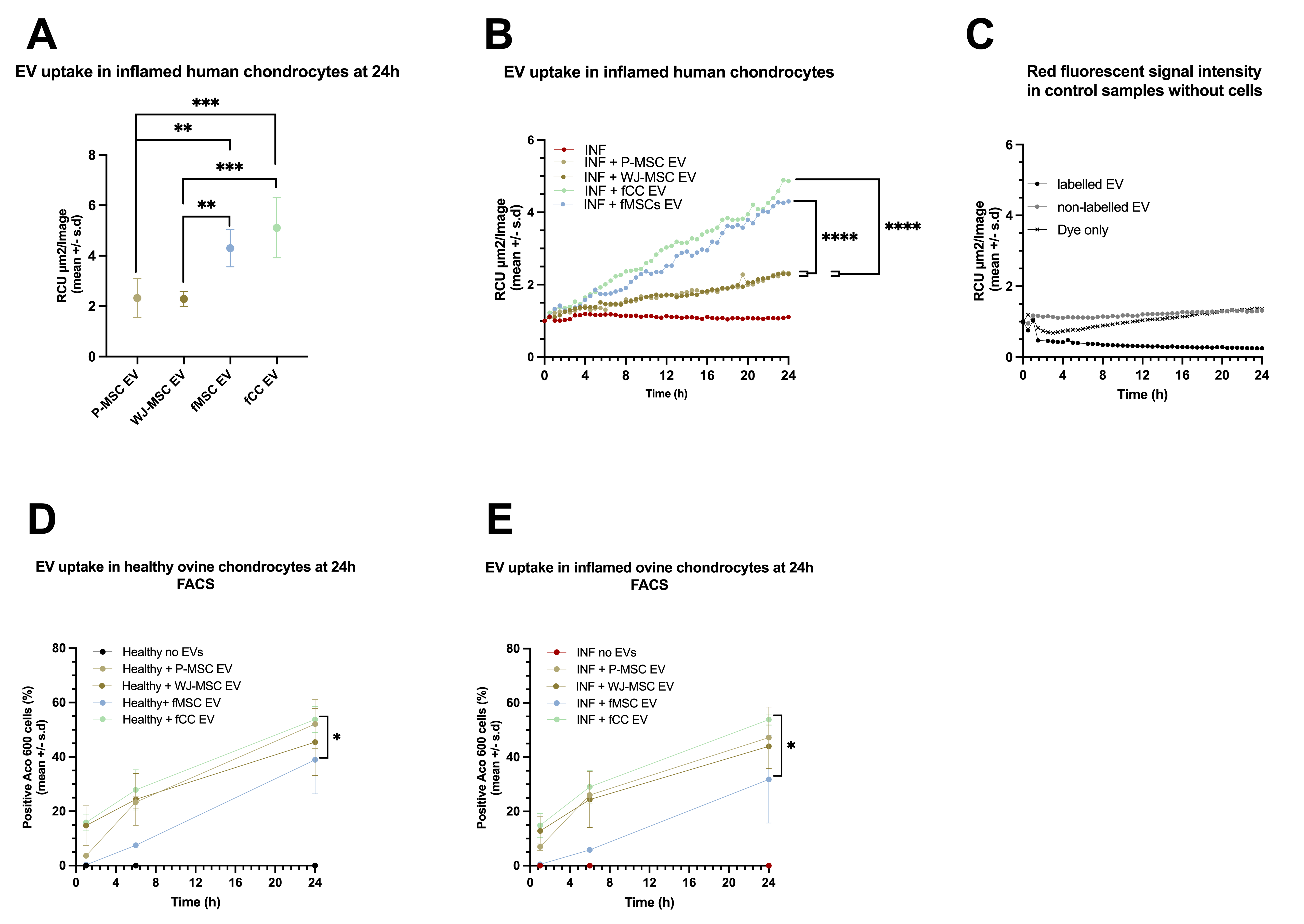

Supplement: Supplementary file 28 — Supplementary Material 28 [file 13287_2025_4585_MOESM28_ESM.tiff]

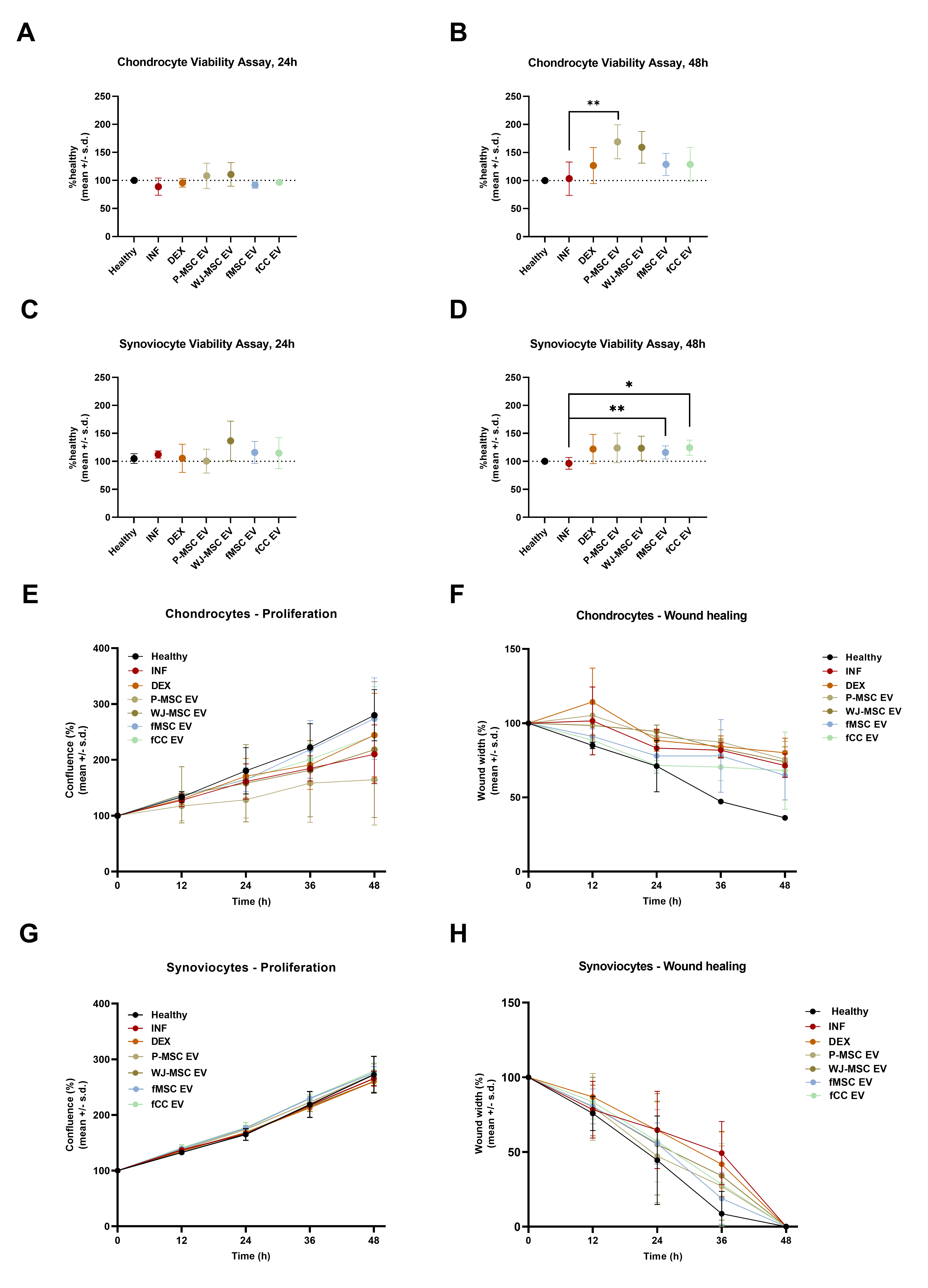

Supplement: Supplementary file 29 — Supplementary Material 29 [file 13287_2025_4585_MOESM29_ESM.tiff]

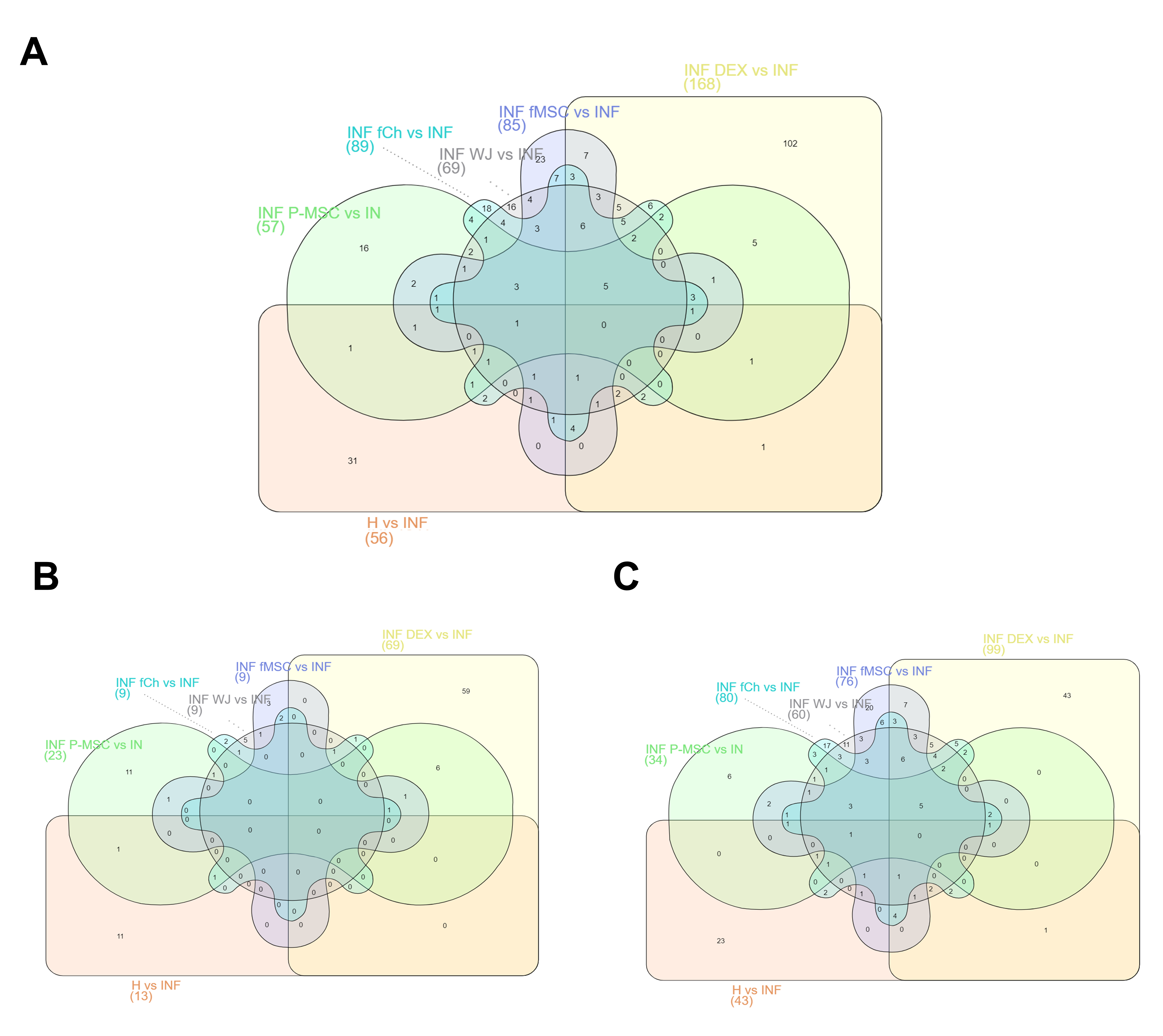

Supplement: Supplementary file 30 — Supplementary Material 30 [file 13287_2025_4585_MOESM30_ESM.tiff]
